# Sour Promotes Risk-Taking: An Investigation into the Effect of Taste on Risk-Taking Behaviour in Humans

**DOI:** 10.1038/s41598-018-26164-3

**Published:** 2018-06-07

**Authors:** Chi Thanh Vi, Marianna Obrist

**Affiliations:** Sussex Computer Human Interaction (SCHI) Lab, School of Engineering and Informatics, University of Sussex, Brighton, UK

## Abstract

Taking risks is part of everyday life. Some people actively pursue risky activities (e.g., jumping out of a plane), while others avoid any risk (e.g., people with anxiety disorders). Paradoxically, risk-taking is a primitive behaviour that may lead to a happier life by offering a sense of excitement through self-actualization. Here, we demonstrate for the first time that sour - amongst the five basic tastes (sweet, bitter, sour, salty, and umami) - promotes risk-taking. Based on a series of three experiments, we show that sour has the potential to modulate risk-taking behaviour across two countries (UK and Vietnam), across individual differences in risk-taking personality and styles of thinking (analytic versus intuitive). Modulating risk-taking can improve everyday life for a wide range of people.

## Introduction

From the earliest days of human history, people have made risky decisions to ensure survival^1^. We know that some humans are more likely to take risks than others^2–4^ and standardized measures accounting for individual differences in sensation seeking^5^ and risk-taking^6^ have been established. In a similar vein, taste is a dedicated warning system that helps people to make important decisions under risk (ingesting or rejecting a given food)^7–10^. Given the parallel between human decision making under risk and the unique properties of the gustatory system^11^, an interaction between risk-taking and the sense of taste can be expected. Recent studies, indeed, demonstrated and validated a correlation between risk-taking personalities and spicy food^12,13^. However, there is no prior knowledge of the relations between the basic tastes and humans risk-taking behaviour. Here we establish for the first time the sour taste as the unique taste quality promoting risk-taking.

Related works^14–19^ suggest that taste has an effect on cognitive processes and decision-making. In particular, Obrist *et al*.^14^ identified different temporal characteristics for the five basic tastes and discussed those differences based on the dual-process theory^20–22^ that accounts for two styles of thinking. The first is the intuition-based System 1 with its associative reasoning that is fast and automatic with strong emotional bonds. The second is the reasoning-based System 2 which is slower and more volatile, being influenced by conscious judgements and attitudes. This prior work suggests that, for instance, based on the explosive but short-lived sensation of a sour taste, people would be left wondering, hence become more rational in their decision-making and act slower. In contrast, the residual characteristic of sweetness coupled with its typically perceived pleasantness (affordance of ingestion) was suggested to stimulate a more intuitive decision-making behaviour and faster actions. Similar to sweet, this prior work suggested that bitter had a tendency to facilitate faster decision-making due to its clear signal of an unpleasant taste (affordance of rejection). However, there were no clear findings suggested for the salty and umami tastes.

In this work, we aim not only to investigate the relationship between the five basics tastes (sweet, sour, bitter, salty, and umami) and human risk-taking behaviour, but also to analyse the temporal differences in their behaviour depending on the taste administered, which has never been reported before, but is motivated by the above cited work^14^. We conducted three between-participants experiments comparing the five taste groups (plus a neutral stimulus) across two countries, UK and Vietnam, accounting for cultural differences in taste perception, in particular of the consumption of MSG, a food additive to create the umami taste^9,23,24^. We used the standardized Balloon Analogue Risk-Taking (BART) task^25^, a computerized gambling task, in our investigation. Each participant ingested only one basic taste and a neutral (water) stimulus in a randomized order across the two blocks of the BART task. Then, participants were asked to pump-up a balloon on a computer screen by clicking the mouse button with the potential either for an accumulated monetary reward or for losing it. After each pump-up action (left mouse click), the balloon increases its size or explodes depending on a randomized algorithm. To win the monetary reward, participants must decide when to stop pumping up the balloon and click ‘cash out’ before the balloon explodes. If the balloon explodes, participants lose all the accumulated money in that trial. (see details in Materials and Methods).

Based on the previously established analytic approach for the BART task^25^, risk-taking behaviour is best measured via the average value of the number of pumps for unexploded balloons (referred to as the ‘adjusted number of pumps’) which has been shown to provide the highest correlation for assessing the risk-taking behaviour. A higher adjusted number of pumps is indicative of greater risk-taking propensity^25^. In addition, we excluded trials with ‘contaminated’ balloons, i.e., balloons used in the neutral stimuli group of the second block of the BART task (see analysis in SI). Thus, we used the total amount of unexploded and uncontaminated balloons as main indicator to determine participants’ risk-taking behaviour. Furthermore, to investigate the specific temporal patterns underlying the decision-making under risk, we analysed the clicking behaviour in the BART task (the time elapsed between pump actions, i.e. the ‘inter-click time’). We used this information as an indication of the participant’s hesitation level when pumping up the balloons (i.e., decide quickly or slowly). We extended this temporal analysis by accounting for both the exploded and unexploded balloons separately, motivated by prior work showing that neural activations when performing the BART task differ between pump-up actions before cashing out or deciding to pump-up more, suggesting that analysing both exploded and unexploded balloons could predict safe or risky choices^26^.

**Figure 1:**
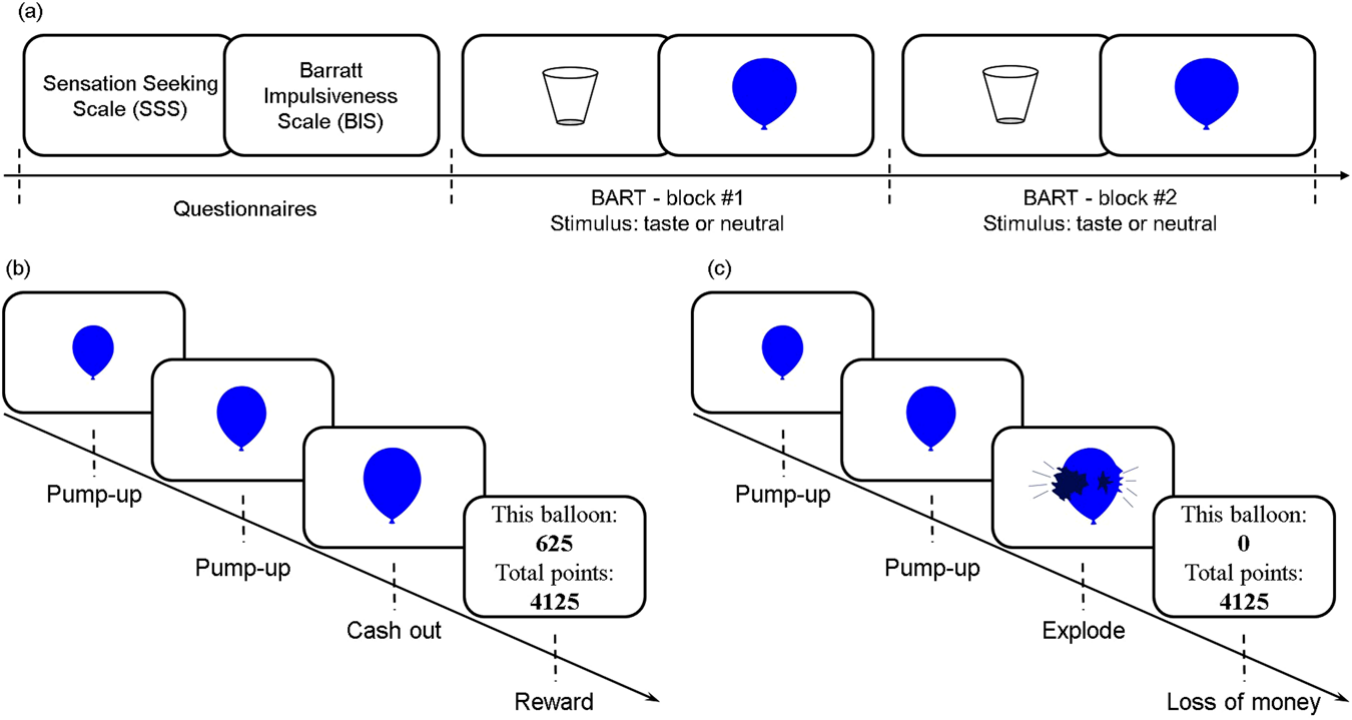
Overview on the experimental procedure. (**a)** Each participant first completed the Sensation Seeking Scale (SSS) and Barratt Impulsiveness Scale (BIS) questionnaires. The results from SSS and BIS were used to counterbalance participants across all five taste groups and ensure equal distribution and avoidance of bias. Each participant then completed two blocks of the Balloon Analogue Risk-Taking (BART) task after ingesting an odourless and colourless taste solution (bitter, sweet, umami, sour, or salty) or a neutral stimulus (mineral water), counterbalanced. Each block consisted of 30 trials and was separated by a short break (5 minutes); (b) and (**c**) illustrate the timeline of a single trial in the BART task, where (**b**) results in a cash out (reward in form of collected points, later converted into money) and (c) results in the explosion of the balloon (loss of all collected points on that trial, and consequently the related money). A new trial started immediately after a participant decided to cash out or if the balloon exploded (see more details in Materials and Methods).

In all three experiments carried out in the UK and Vietnam, we controlled for the distribution of high and low risk-taker personalities in the taste groups by using two standardized questionnaires (Sensation Seeking Scale - SSS^27^ and Barratt Impulsiveness Scale - BIS^6^). Figure 1 shows an overview on the procedure, where each participant ingested either one of the five basic tastes (sweet, bitter, sour, salty, or umami) or a neutral stimulus (mineral water), counterbalanced, before performing each of the two blocks of the BART task. (see more analysis details in the Supplementary Material).

## Results

### Sour promotes risk-taking behaviour

**Figure 2:**
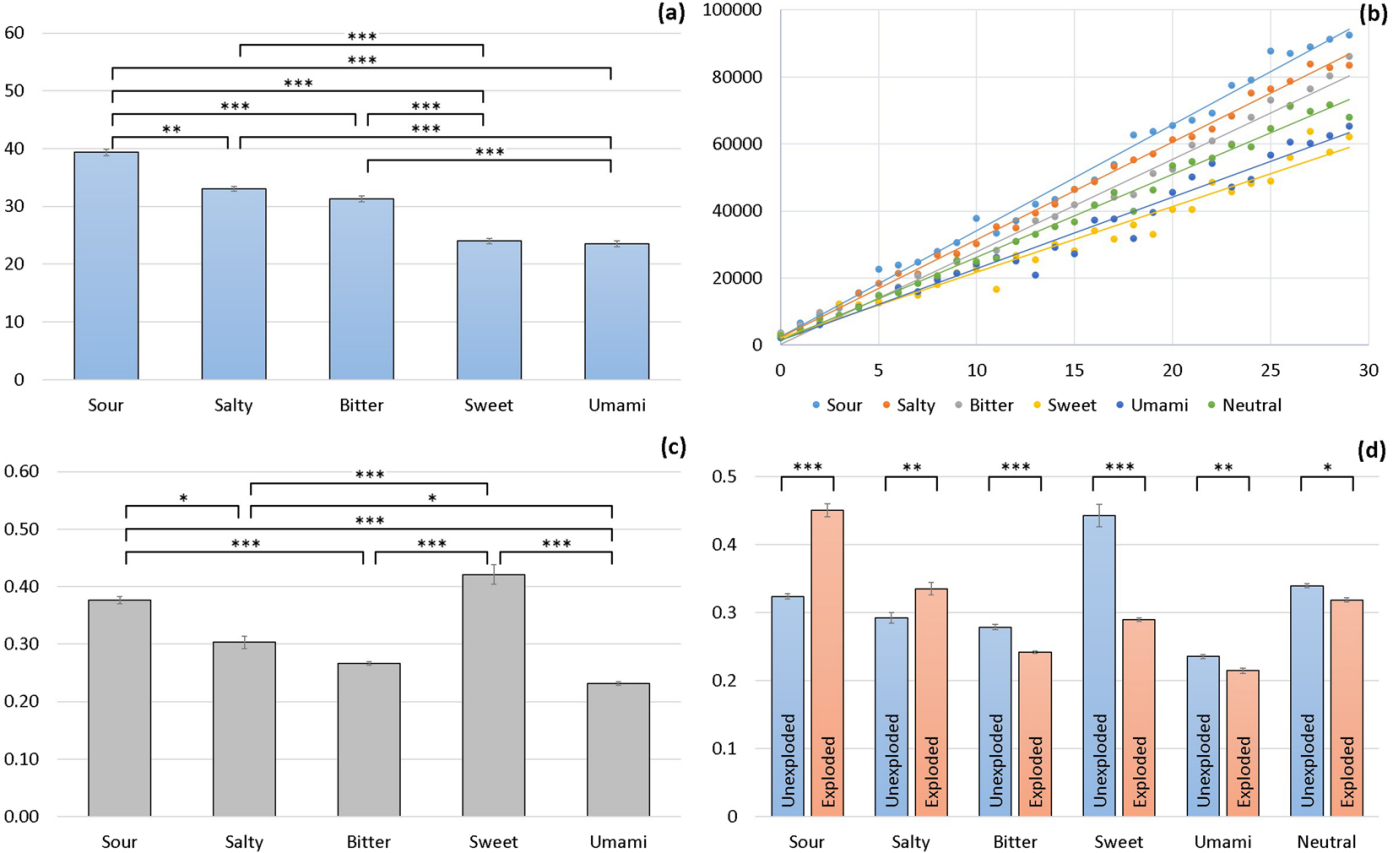
Experiment 1 results with 70 UK participants. **(a)** The average adjusted number of pumps for unexploded and uncontaminated balloons indicating risk-taking behaviour across all 5 basic taste groups (**p < 0.01, ***p < 0.001). **(b)** Accumulated cash reward (in points, vertical axis) earned by participants in each stimulus group (uncontaminated trials) in a time-course of a block of 30 trials (horizontal axis). **(c)** The average inter-click time between pump-up actions for uncontaminated (both exploded and unexploded) balloons across all five stimulus groups. **(d)** Average inter-click time (in seconds) across all trials, organized by stimulus group and by unexploded and exploded balloons. Pairwise comparisons found significant differences in all stimuli and between exploded and unexploded balloons (*p < 0.05, **p < 0.01, ***p < 0.001). Bars represent standard error of the mean (SE).

In the first experiment (see Fig. 1), we collected data from seventy participants in the UK (46 females, mean age 25.0 ± 6.4). Each participant performed two blocks of the BART task after ingesting one of the five basic tastes or mineral water (neutral solution). Analysing the unexploded and uncontaminated balloons, our results show that (i) ‘sour’ promoted high risk-taking behaviour, (ii) ‘sweet’ and ‘umami’ supported low risk-taking behaviour; and (iii) ‘bitter’ and ‘salty’ triggered neither risky nor safe behaviour. Fig. 2(a) provides an overview of the average adjusted number of pumps for unexploded and uncontaminated balloons as key indicator for participants’ risk-taking behaviour. On average, participants who ingested sour pumped the balloons 39.36 times for unexploded balloons. This is significantly higher than with any other taste (39.08% more than sweet, 20.50% more than bitter, 16.03% more than salty, and 40.29% more than umami). In other words, participants aimed to maximize their cash reward by taking greater risks with the sour taste (see Fig. 2(b) for the accumulated money (represented in points, vertical axis) over time for each stimulus in the first experiment).

Analysing the participants pumping behaviour in more detail, our results showed that the sweet, followed by the sour taste, made participants hesitate the most (decide slower). Figure 2(c) shows that the inter-click time for pumping up balloons in the sour group was significantly higher (M 0.38 seconds SE 0.01) compared to the salty (M 0.30 seconds SE 0.02), bitter (M 0.27 seconds SE 0.01), and umami (M 0.23 seconds SE 0.01) groups, but similar to the sweet group (M 0.42 seconds SE 0.03). When further comparing pairs of exploded versus unexploded balloons in terms of their inter-click times, we found significant differences between the taste groups. Participants in the sour and salty group exhibited significantly (p < 0.001) greater inter-click times on pumping up exploded balloons than on unexploded balloons. Figure 2(d) shows that this behaviour was reversed for bitter, sweet and umami, as well as for the neutral stimulus, where participants exhibited greater inter-click times on the unexploded balloons. In summary, from this first exploratory experiment, we learned that the effect of the five basic tastes on risk-taking behaviour is divided into three clusters, where sour promotes the highest and sweet and umami the lowest risk-taking, while no clear picture was obtained for bitter and salty.

### Sour promotes risk-taking across two countries

**Figure 3:**
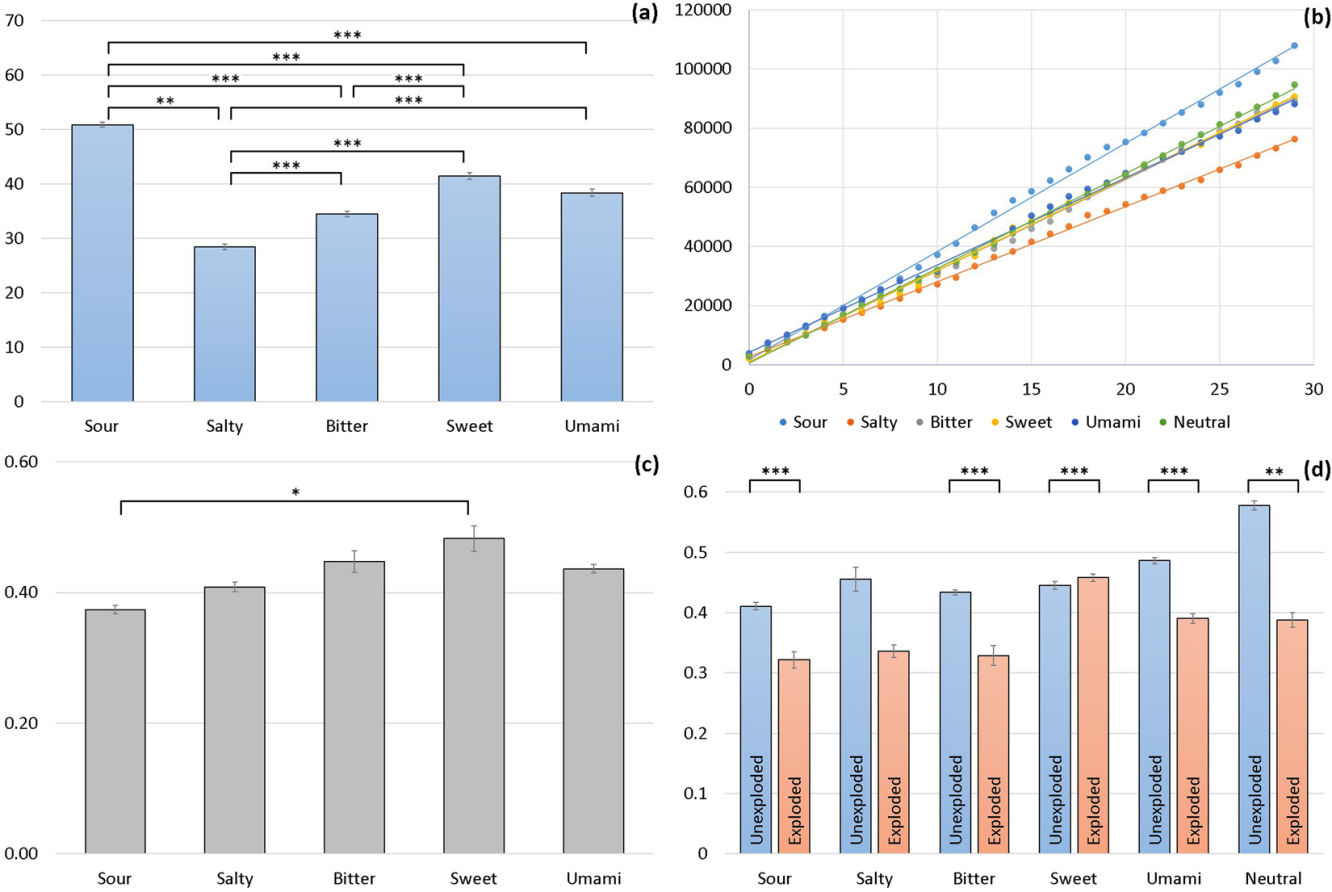
Experiment 2 results with 71 Vietnamese (VN) participants. **(a)** The average adjusted number of pumps for unexploded and uncontaminated balloons) indicating risk-taking behaviour across all 5 basic taste groups (**p < 0.01, ***p < 0.001). **(b)** Accumulated cash reward (in points, vertical axis) earned by participants in each stimulus group (uncontaminated trials) in a time-course of a block of 30 trials (horizontal axis). **(c)** The average inter-click time between pump-up actions for uncontaminated (both exploded and unexploded) balloons across all five stimulus groups. **(d)** Average inter-click time (in seconds) across all trials, divided by stimulus group and by unexploded and exploded balloons. Pairwise comparisons found significant differences in all stimuli and between exploded and unexploded balloons (**p < 0.01, ***p < 0.001). Bars represent standard error of the mean (SE).

To further understand the effect of taste on risk-taking and account for known cultural differences in taste perception, in particular for the umami taste^9,23,24^, we repeated the same experiment with an identical procedure and sample size (N = 71) in Vietnam (see Fig. 3). Vietnam has the 3rd largest mono-sodium glutamate (MSG) consumption^28^ (MSG is a compound used to present the umami taste). Despite being rich in protein and nutritious, umami in its pure form is often perceived as unpalatable in Western countries. Due to the higher consumption of MSG-rich food in Asian countries^28^, a different perception and reaction could be expected. We hypothesized that the low risk-taking effect of umami would be confirmed, but that the temporal pattern would change from the fastest inter-click time in the UK sample, to a slower inter-click time, and consequently closer to sweet. This was based on the assumption that the perceived pleasantness of the taste changes across the two countries and modulate the effect in the BART task.

The results from the second experiment confirmed the sour taste as the unique taste quality promoting the highest risk-taking behaviour. Figure 3(a) shows an overview on the average adjusted number of pumps for unexploded and uncontaminated balloons and (b) accumulated monetary reward (represented in points). While, those results confirmed sour in promoting the highest risk-taking behaviour (M 50.88 ± 0.99), no confirmation was obtained for the other four tastes, which can be arranged into an increasing order (from lowest to higher risk-taking behaviour): salty (M 28.46 ± 1.03), bitter (M 36.00 ± 0.98), umami (M 38.37 ± 1.33), and sweet (M 41.47 ± 1.25). Surprisingly, in contrast to the UK sample, both sweet and umami changed to promote riskier decisions.

We explored the temporal patterns, analysing the inter-click time between pump-up actions for the Vietnamese participants. Figure 3(c) shows that sour exhibited, to our surprise, the fastest inter-click times (M 0.37 seconds SE 0.01) in contrast to the results in the first experiment, were the inter-click time for sour was - after sweet - the slowest amongst the tastes. Interestingly, however, despite this different positioning of sour in the range of the other tastes, there was no significant difference between its inter-click times for the two experiments (i.e., 0.38 versus 0.37 seconds in the UK and Vietnam, respectively). That means, that sour not only remains the unique taste quality to promote risk-taking but the sour effect on users’ performance (inter-click time) is stable across two countries (different cultural contexts).

When analysing the exploded and unexploded pumping up actions separately, the results from the second experiment show that participants exhibited significantly different inter-click times for balloons that were going to be cashed out (unexploded balloons) than for those that were going to explode. This was true for all tastes but sweet, where no significant difference was found. With respect to sour, we can see a difference between the UK and Vietnamese sample with respect to sour to the extent that the UK participants exhibited significantly greater inter-click times on the exploded balloons (Fig. 2(d)), while the Vietnamese participants exhibited greater inter-click times on the unexploded balloons (Fig. 3(d)). This distinction could be explained through differences in the strategies applied by the participants in the two samples (please note that the UK sample was more diverse with respect to the participants background compared to the Vietnamese sample, which was fully recruited from the School of Biotechnology and Food Technology, Hanoi University of Science and Technology).

Taken together, the results from both the first and second experiment provide compelling evidence for sour in promoting riskier behaviour across two countries and risk-taking personalities (in both experiments we controlled and balanced low and high risk-takers). To account for individual differences in strategies when completing the BART task, we designed a third experiment controlling for the differences in the individual participants’ strategies and decision making (i.e., style of thinking).

### Sour promotes risk-taking in analytic and intuitive thinkers

**Figure 4:**
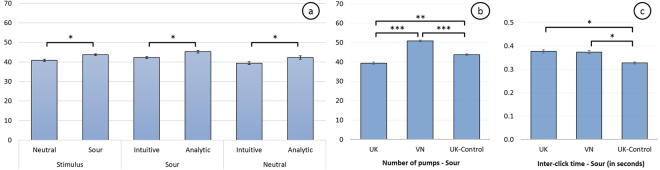
Experiment 3 (UK-Control) results and comparison to the results from the first (UK) and second (VN) experiments: **(a)** Average adjusted number of pumps for unexploded and uncontaminated balloons for each stimulus (neutral and sour) accounting for intuitive and analytic thinkers based on the Cognitive Reflection Test (CRT); **(b)** Comparison of the adjusted number of pumps for unexploded and uncontaminated balloons of sour in three experiments: 70 UK participants (experiment 1), 71 Vietnamese participants (VN - experiment 2), 16 analytic vs. 11 intuitive participants (UK-Control - experiment 3); **(c)** The same comparison with the inter-click times representing the hesitation level. Bars represent standard error of the mean (SE).

In the third experiment (see Fig. 4), we explicitly informed participants about the average explosion point in the BART tasks (64 pumps), hence reducing the level of uncertainty in their decision making. Each participant performed two blocks of the BART task and was presented either with a sour stimulus or mineral water, in a counterbalanced order. We expected participants to align their strategies in the pumping up actions, reflected in an increase in the risk-taking behaviour in both conditions. Additionally, we controlled for individual differences in style of thinking (i.e., intuitive versus analytic). We used the Cognitive Reflection Test (CRT)^29^ and marked participants who answered two or more from the three questions correctly as “analytic”, whereas participants who answered less than two questions correctly were marked as “intuitive”^29^. We hypothesised that participants applying an analytic approach would make riskier choices to get a higher reward in comparison to intuitive thinkers.

As before, sour again significantly promoted risk-taking behaviour compared to the neutral stimulus (p < 0.05). Figure 4(a) shows that the average adjusted number of pumps for unexploded and uncontaminated balloons was significantly higher for the sour group (M 43.76 SE 0.85) compared to the neutral stimulus (M 40.83 SE 1.13).

Participants in both sour and neutral conditions exhibited the same distinct risk-taking behaviour. Participants following an analytic style of thinking took significantly more risk (M 43.77 SE 1.05) compared to participants following an intuitive style of thinking (M 40.80 SE 0.93, p < 0.05). These results suggest that sour promoted riskier choices regardless of the style of thinking. It remains to be explored as to how the style of thinking is correlated with a persons’ risk-taking personality.

Moreover, when the uncertainty in the BART task was reduced (participants in the 3rd experiment - UK-Control were informed about the average explosion point), our results show that user’s performance significantly improved compared to the first (13% faster) and second experiment (11% faster) (Fig. 4(c)). These results indicate that participants in experiment 3 performed faster under reduced uncertainty, while sour consistently promoted riskier decisions across all three experiments (see Fig. 4(b) for comparison between the three experiments). Additionally, our analysis of the inter-click time showed that the inter-click time slowly decreased over time across all taste stimuli and in all three experiments (linear regression slope in Experiment 1 is: M -0.0062 SD 0.0012, in Experiment 2 is: M -0.0059 SD 0.0015, in Experiment 3 is: M -0.0037 SD 0.0003). This result is similar to previous work that analysed inter-click time in each trial of the BART task synchronized with the captured EEG signals (by analysing the amplitude of the P300^30^). In this prior work, it has been shown that the slow decrease was due to participants’ hesitation at the beginning of the BART task. As participants progressed, they established the reward structure of the task and were clicking faster^30^.

## Discussion

The chief finding of the present work is the identification of the sour taste as the one basic taste that consistently promotes a riskier behaviour. Our findings show that sour modulates risk-taking across two countries, individual differences in risk-taking personalities and styles of thinking.

One might now ask why, in the real world, would anyone want to promote a riskier behaviour of people? Risk-taking is part of our everyday life and without taking risks, humans do not learn new skills or learn to cope with situations in uncertain circumstances^31^. Our findings suggest that, at least in the context for the BART task involving potentially winning small amounts of money, sour does not provoke people to indulge in reckless risky habits, but has unique attributes to modulate risk-taking and may encourage risk-averse people to take new opportunities and potentially lead to a happier life. For example, people who are risk-averse (e.g., people with anxiety disorders or depression) may benefit from a sour additive in their diet. Knowing that sour promotes a riskier behaviour, it allows us to explore suggestions to either promote or inhibit risk-taking behaviour through employing a sour-reduced or sour-enriched diet. Thus, instead of avoiding risk, sour could reinforce a desired behaviour without putting people at risk and encourage, especially risk-averse people, to take new opportunities and support self-improvement (e.g., to leave the house or talk to a stranger). Prior work has, for instance, shown that in cases of psychiatric disorders such as depression, anxiety, or stress-related disorders the use of lemon oils proved efficient^32^ and was further demonstrated to reduce stress^33^. While lemon and sour are not the same, they share common properties that can be further investigated with respect to risk-taking. Similarly, our sense of taste is not only one of the main human sensory systems, evolved to modulate human feeding behaviours^34^, by guiding food ingestion (acceptance versus rejection) and metabolization^10^, but taste has also a strong link with human affections (punishment and reward)^8,35^, social behaviours (sweet taste preferences and experiences predict personalities and behaviours)^36^, and cognitive processes (e.g., the amygdala, the prefrontal cortex, and the insular cortex are the candidates for the interactions between the taste system and the reward and feeding system^35^).

Although more research is needed to fully understand the human gustatory system, amongst other underlying detection mechanism of sour in taste cells^37–39^, we are convinced that this work provides a rich space for other researchers to develop and test new hypotheses, including the prediction of neural activations on the effect of sour^26^. Further explorations of the cross-cultural effect of the sour taste on risk-taking (e.g., studying additional cultural groups) and for specific user groups can be envisaged^31^. These explorations will consequently strengthen this line of research and push the results a step forward into a real world context. Furthermore, although we measured each participant’s liking reaction for the ingested taste stimuli, it would be interesting to monitor any changes in participant’s emotional state or liking as a result of the ingested taste^40^ during the BART task. This is particularly relevant to real-world cases such as using flavoured chewing gum^41^ or taste candies^42^ instead of using taste solution.

Finally, while the design of future taste-based technologies is increasingly fascinating engineers, computer scientists, and designers of novel user interfaces, we still lack a comprehensive understanding on how such new technologies are going to affect users’ performance and human behaviour. We believe that our findings can inspire and steer the design of novel gustatory interfaces^43–46^. This work provides a first but essential step towards decoding the effect of taste on human decision-making under risk.

## Methods

### Subjects

Seventy participants in the UK (46 females, 24 males; mean age 25.0 ± 6.8), seventy-one participants in Vietnam (45 females, 26 males; mean age 20.2 ± 1.72), and twenty-seven participants in the UK (22 females, 5 males; mean age 22.22 ± 3.96) volunteered to participate in the first, second, and third experiments respectively. Participants were recruited from the local institutions, mainly representing students and staff. Their demographic details are included in the SI. In our study, we did not control for gender balance in each taste group as prior work has shown that there is no significant influence of gender on emotional responses to basic tastes^47^. Instead, we controlled for individual differences in risk-taking behaviour in each experiment, using two standardized questionnaires (i.e. sensation seeking (SSS) and impulsiveness (BIS) questionnaires) before commencing with the experiment. Participants were asked through self-report to confirm that they did not have food allergies, and neither were a smoker nor were pregnant. They were asked to refrain from eating for at least 2 hours before participating in the experiment. Participants also confirmed that they did not suffer from any sensory dysfunction (e.g., Dysgeusia, a taste disorder). All participants were free of psychotropic medications and had normal or corrected-to-normal vision. Each participant gave written informed consent after the explanation of the experiment protocol, as approved by the Life Sciences & Psychology Cluster-based Research Ethics Committee (C-REC), University of Sussex. All experiments were performed in accordance with relevant guidelines and regulations.

### Selection of stimuli

The stimuli were presented as odourless and colourless water solution in a disposable plastic cup (30 ml). The tastant/water volume provided to participants each time was 20 ml. Participants were informed that the provided solution might contain one of the five basic tastes (bitter, sour, salty, sweet, and umami), or just water^48^. All stimuli were prepared and delivered at room temperature, at about 23 °C. Details of the chemicals used and concentration for each of the 5 taste stimuli plus water as neutral stimulus are: Bitter - Caffeine (5 mM)^49,50^, Salty - Sodium Chloride (90 mM)^48,50,51^, Sour - Citric Acid (10 mM)^52^, Umami - l-glutamic acid monosodium salt hydrate^48^ (50.0 mM), Water – Evian mineral water^48^.

### Task

We performed a between-subjects design where each participant ingested only one basic taste and a neutral (water) stimulus in a randomized order across the two blocks in the experiment. In all three experiments, participants were asked to complete two questionnaires. Participants in the third experiment were additionally asked to complete the Cognitive Reflection Test (see details below). They then performed two blocks of the BART task where the monetary reward correlated with their risk-taking behaviour. Before each block, they were asked to ingest a 20 ml solution presented in a 30 ml plastic cup. During the experiments, participants followed instructions provided on a computer screen.

Upon arrival, participants were given time to read the information sheet explaining the procedure and details of the experiment. Participants were encouraged to ask any questions before signing the consent form. Participants of the first and second experiment then went through a pre-screening process where they completed two questionnaires to measure their two personality traits: the Sensation Seeking Scale (Q1) and the Barratt Impulsiveness Scale (Q2).

Q1: The Sensation Seeking Scale (SSS)^27^ was used to measure the sensation seeking of a participant. This scale contains 40 questions with 4 different aspects (thrill and adventure seeking – TAS, disinhibition – Dis, Experience Seeking – ES, and Boredom Susceptibility – BS). A total score (in the range of 0–40) was calculated at the end of the questionnaire and sent to the experimenter. Participants were not aware of their score at this stage.

Q2: The Barratt Impulsiveness Scale (BIS)^6^ is a widely used self-report measure of impulsive personality traits. It includes 30 items that are scored to yield six first-order factors (attention, motor, self-control, cognitive complexity, perseverance, and cognitive instability impulsiveness) and three second-order factors (attentional, motor, and non-planning impulsiveness). Similar to the previous questionnaire, a total score (in the range of 0–120) was given to the experimenter at the end of the questionnaire and not shown to the participant.

Participants of the third experiment were additionally asked to complete the Cognitive Reflection Test (Q3) before proceeding with the SSS and BIS questionnaires.

Q3: The Cognitive Reflection Test (CRT) is a short psychological task composed of three questions, developed by Shane Frederik^29^. CRT accounts for two general types of cognitive activity. The first is executed quickly without reflection, the latter requires conscious thought and effort. These are labelled “system one” (intuitive thinking) and “system two” (rational thinking) following the dual process theory^20,21^. The CRT consists of three questions that each have an obvious response that activates system 1, but which is incorrect. The correct response requires the activation of system 2. However, for system 2 to be activated, a person must note that their first answer is incorrect, which requires them to reflect upon their own cognition. The test was given to participants in the paper form and was collected by the experimenter immediately after it was done. The experimenter did not reveal to participants of how many answers were correct or which group they were assigned to (intuitive or analytic) until the end of the experiment.

Participants completed the questionnaires with a 2-minute break between them. Q1 and Q2 and their instructions were coded into a computer program (using Microsoft Visual C#), whereas Q3 was presented on paper. For Q1 and Q2, the program showed one multiple-choice question at a time and only advanced to the next question once the participant had made their choice. The scores of SSS and BIS questionnaires (Q1 & Q2), according to each participant, were used for assigning participants to one of the five taste groups (bitter, salty, sour, sweet, and umami) in the following part of the experiment. Within each group, participants experienced one taste in one block and one neutral stimulus (water) in another block, in a counterbalanced order. They were instructed to rinse their mouth with the taste solution before swallowing it (‘sip and swallow’ approach). Participants within each group were similar in terms of SSS and BIS scores to remove any bias of one group having a higher number of risk-taking participants than another group. The scores of the CRT questionnaire (Q3) were used for assigning participants to either Intuitive group (with correct answers less than 2–16 participants) or Analytic group (with correction answers greater or equal to 2–11 participants).

### Data availability statement

The datasets generated during and/or analysed during the current study are available from the corresponding author on reasonable request.

## Electronic supplementary material

Video

Supplementary information
