# Supplementary material for: Sour Promotes Risk-Taking: An Investigation into the Effect of Taste on Risk-Taking Behaviour in Humans
Source: Sci Rep. 2018 Jun 7;8:7987. doi: 10.1038/s41598-018-26164-3 (PMC5992179; doi:10.1038/s41598-018-26164-3)
Supplement: Supplementary file 2 — Supplementary information [file 41598_2018_26164_MOESM2_ESM.pdf]

Sour Promotes Risk-Taking: An Investigation into the Effect of Taste on Risk-Taking Behaviour in Humans

Chi Thanh Vi[1] and Marianna Obrist[1]

[1]Sussex Computer Human Interaction (SCHI) Lab, School of Engineering and Informatics, University of Sussex, UK

First Experiment - United Kingdom (UK)

We collected data from 70 participants (46 females, 24 males), who were staff and students from the local university. We collected 4,200 trials of the BART task (70 participants x 2 blocks x 30 trials).

First, to ensure the risk-taking personalities between stimuli are balanced, we looked at their captured SSS and BIS scores of participants belong to these groups (see Figure S1). Multivariate ANOVA with Bonferroni correction on these scores showed no significant difference between taste groups (p=1.000), and between orders (p=0.978). We also found no interaction between the order of taste with SSS scores (p=1.000), as well as with BIS scores (p=0.921). This ensured a balanced distribution of risk-taking nature within participants across all taste groups.

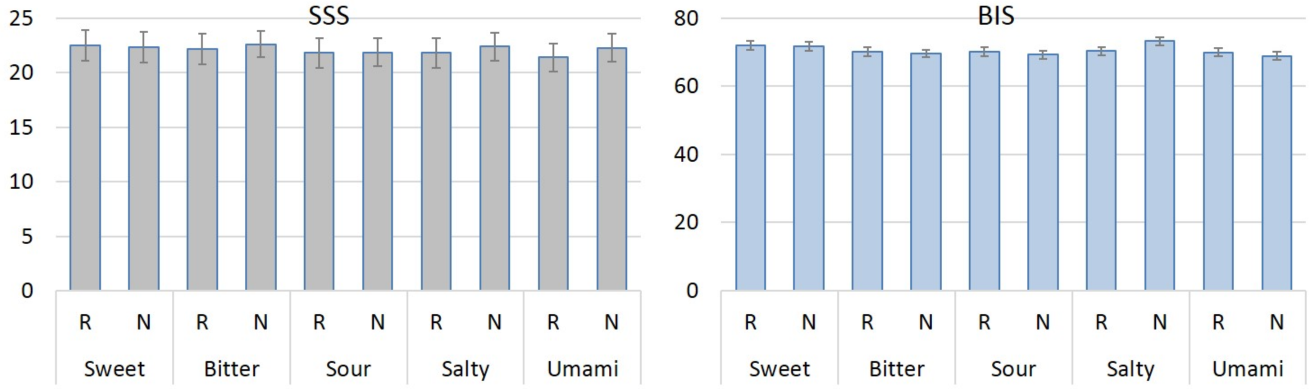

Figure S 1. Sensation Seeking Scale (SSS - top) and Barratt Impulsiveness Scale (BIS - bottom) scores of different taste groups, in two orders (R - reverse order: neutral then taste, and N - normal order: taste then neutral). Bars represent standard error of the mean (SE).

Among the 4,200 collected trials, we only considered the unexploded balloons. Thus, we removed 1,194 trials where the balloon exploded, accounting for 28.5% of total trials. Because each participant performed two blocks of the BART task, one block with a taste and another with the neutral stimuli, we separated the trials into six groups of five tastes and the neutral stimulus.

We analysed the adjusted number of pumps across balloons (i.e., BART score). We performed a univariate ANOVA with Bonferroni correction to identify significant differences between the taste groups as well as any interaction between the tastes and the order. We found a significant difference between taste groups and there was interaction between Taste*Order. To further investigate the order effect, we performed post-hoc tests with the neutral stimuli for each taste group, comparing the task performance (adjusted number of pumps) between the first block and the second block of the BART task. The results show significant differences within the order of neutral stimulus in Bitter ($p<0.01$), Sour ($p<0.001$), and Umami ($p<0.05$) (see Figure S2 and Table S1).

These results indicate that the neutral stimuli in each taste group might already be 'contaminated' through the taste administered in the first block. Therefore, we removed all trials where participants had the neutral stimulus in the second block, accounting for the carry-over effect. Following this, we removed 743 neutral trials, equivalent to 16.65% of all unexploded balloons. Figure S3 shows the percentages of the unexploded balloons for each taste group, before and after the removal of 'contaminated' balloons. We then divided the trials into 6 groups (5 tastes and 1 neutral).

For those 6 groups (5 tastes and 1 neutral), we then compared risk-taking behaviour based on the adjusted number of pumps (unexploded balloons). Using this as the dependent variable, we performed repeated measure ANOVA with Bonferoni

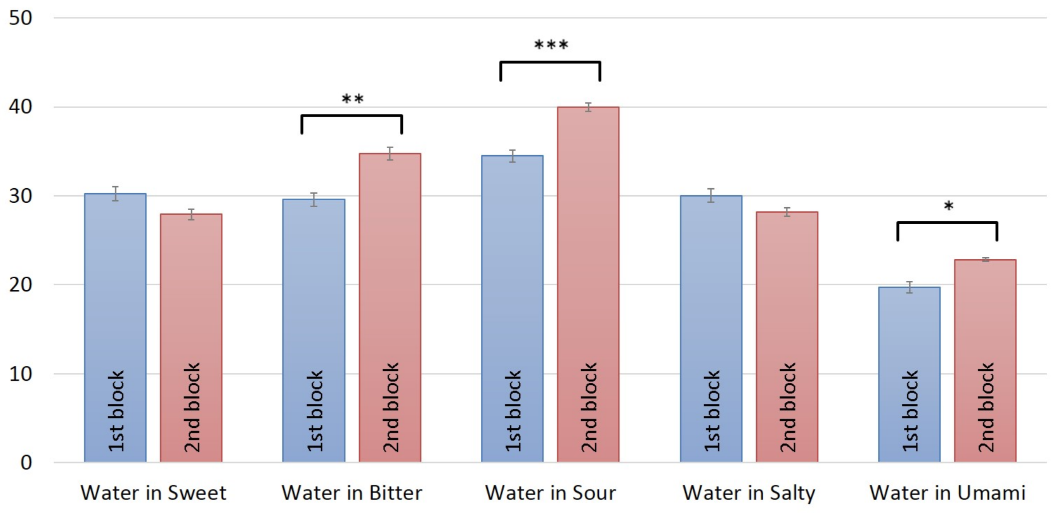

Figure S 2. Post-hoc comparison of adjusted number of pumps per unexploded balloon within neutral stimuli for each taste, between the first block and the second block (*p<.05, **p<0.01, ***p<.0001). For example, for the sweet taste we compared the first block, where participants had water before having sweet, to the second block, where participants had water after having sweet. Bars represent standard error of the mean (SE).

Table S 1. Summary of adjusted number of pumps (± SE) for the neutral stimuli in the 1st block and the 2nd block of each taste.

	Water in Sweet	Water in Bitter	Water in Sour	Water in Salty	Water in Umami
First block	30.25 ± 1.54	29.59 ± 1.48	34.47 ± 1.33	30.03 ± 1.53	19.71 ± 1.21
Second block	27.91 ± 1.36	34.76 ± 1.23	39.97 ± 1.45	28.15 ± 1.00	22.83 ± 0.95

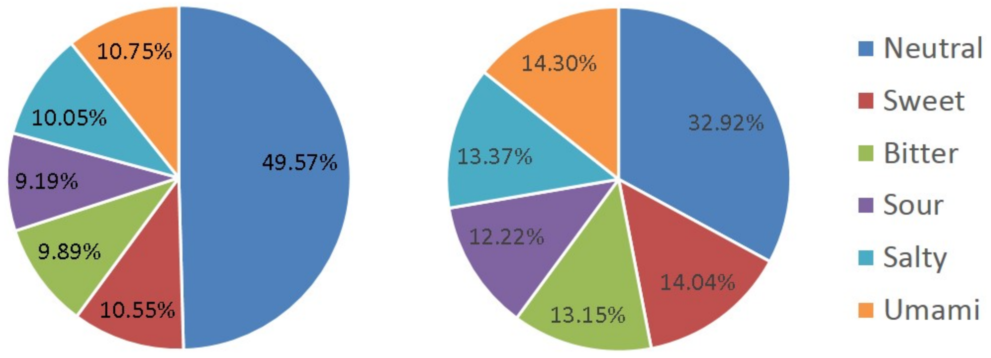

Figure S 3. Percentage of the unexploded balloons for each taste group, with the contaminated neutral stimuli (left), and uncontaminated neutral stimuli (right).

correction on each taste group comparing Neutral vs. Taste to determine the within subject effect in each group. We found the main effect of Taste in the groups of sweet ($F_{1,137}$ = 16.95, p<0.001), sour ($F_{1,143}$ = 23.92), and umami ($F_{1,166}$ = 34.27). We then performed an one-way ANOVA with Bonferroni correction to determine the between subject effect of the five taste groups. We found significant differences within the group ($F_{4,1505}$ = 43.73, p<0.001). Post-hoc tests show that the five taste groups can be clustered into three main categories, based on their risk-taking induced effect: (i) sweet, umami; (ii) bitter, salty; and (iii) sour. We found significant differences in all comparisons between pairs of taste groups but not between bitter vs. salty (p=1.0), sweet vs. umami (p=1.0) (see Figure S4 for illustrations of the average values and post-hoc comparisons, and Table S2 for precise summary values). In terms of gender, male participants performed significantly higher (p<0.001) number of pump (M

38.53 SE 0.80) across all stimuli, comparing to female participants (M 25.68 SE 0.42).

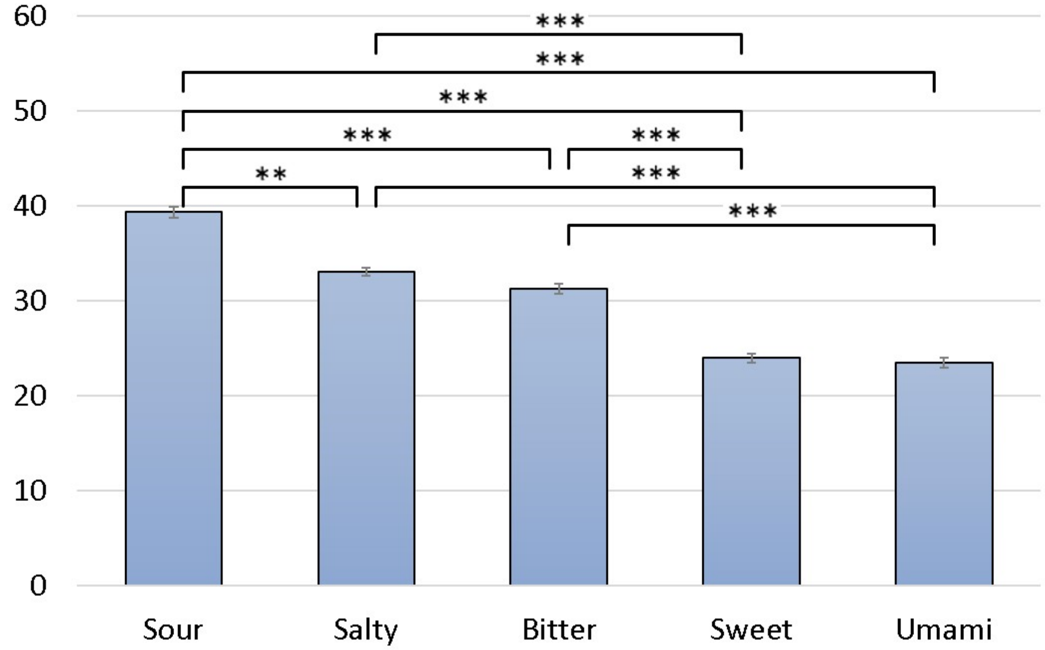

Figure S 4. Average adjusted number of pumps indicating risk taking behaviour (**p<0.01, ***p<0.001). Bars represent standard error of the mean (SE).

Table S 2. Summary of adjusted number of pumps for unexploded and uncontaminated balloons.

	**Sour**	**Salty**	**Bitter**	**Sweet**	**Umami**	**Neutral**
Reverse	39.54 $\pm$ 1.42	37.58 $\pm$ 1.20	28.11 $\pm$ 1.14	31.96 $\pm$ 1.68	25.03 $\pm$ 1.68	28.88 $\pm$ 0.73
Normal	39.15 $\pm$ 1.79	28.78 $\pm$ 0.96	34.03 $\pm$ 1.60	17.45 $\pm$ 0.94	22.02 $\pm$ 1.17	
All balloons	39.36 $\pm$ 1.13	33.05 $\pm$ 0.80	31.29 $\pm$ 1.02	23.99 $\pm$ 1.00	23.50 $\pm$ 1.02	28.88 $\pm$ 0.73

Influence of the stimuli's pleasantness

To investigate the influence of the stimuli's pleasantness on risk-taking behaviour, we calculated the correlation between the rating of stimulus pleasantness (given by participants right after ingesting the stimulus) and the adjusted number of pumps for that stimulus' block. The Pearson correlation test also shows no correlation between the pleasantness ratings of the stimuli and the adjusted number of pumps (r=-0.069, p=0.49). Specific for each individual stimulus, we also found no correlation in neutral (r=-0.18, p=0.30), sweet (r=0.18, p=0.53), bitter (r=-0.23, p=0.44), sour (r=-0.26, p=0.37), salty (r=0.01, p=0.97), and umami (r=0.52, p=0.0536). Participants rated sweet slightly pleasant (mean 1.43 $\pm$ 0.62), bitter slightly unpleasant (-1.29 $\pm$ 0.59), sour as neither pleasant nor unpleasant (0.0 $\pm$ 1.07), salty slightly unpleasant (-0.57 $\pm$ 1.05), umami slightly unpleasant (-0.71 $\pm$ 0.70), and neutral slightly pleasant (0.53 $\pm$ 0.75).

Participant behaviour over time

To investigate the temporal pattern of participants' performance in the six groups, we analysed clicking behaviour in the BART task (the time elapsed between the current pump and the previous pump). First, we aligned all trials with the first click to pump up the balloon. We then averaged all trials of all participants for each taste, and for exploded/unexploded balloons separately. We then performed repeated measure ANOVA with Bonferoni correction on each taste group comparing Neutral vs. Taste to determine the within subject effect in each taste group. We found the main effect of Taste in the groups of bitter ($F_{1,97} = 29.95$, $p<0.001$) and umami ($F_{1,89} = 39.71$). We then performed an one-way ANOVA with Bonferroni correction to determine the between subject effect of the five taste groups. We found significant differences within the group ($F_{4,457} = 20.22$, $p<0.001$). Post-hoc tests show significant differences in all comparisons between pairs of taste groups but not in pairs of (bitter vs. salty),

(bitter, umami), (sour, sweet) with p>0.05. Figure S5 illustrates the average inter-click time for a pump-up action of both exploded and unexploded balloons, divided by the six stimuli, and Table S3 shows the precise values.

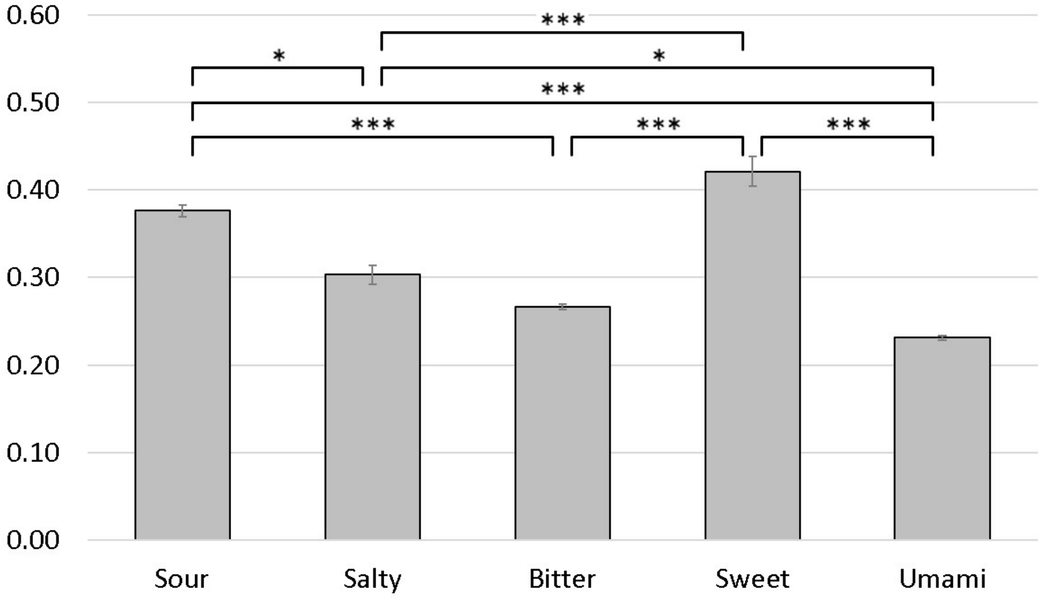

Figure S 5. The average inter-click time between pump-up actions for uncontaminated balloons, divided into five taste groups (* $p<0.05$, ** $p<0.01$, *** $p<0.001$). Bars represent standard error of the mean (SE).

Table S 3. Average time participants spent pumping up balloons, divided by 5 basic taste stimuli.

	**Sour**	**Salty**	**Bitter**	**Sweet**	**Umami**
M	0.38	0.30	0.27	0.42	0.23
SE	0.01	0.02	0.01	0.03	0.01

In addition, we looked into details of exploded and unexploded balloons separately. Figure S6 and Table S4 show the average time spent for a pump-up action, averagely over all trials of each stimulus, and separately between exploded and unexploded balloons. Pair-wise comparisons showed significant difference between them in all stimuli. Specifically, participants spent significantly more time pumping up balloons that were going to be cashed out than the ones that were going to explode in the sweet ($p<0.001$), bitter ($p<0.001$), salty ($p<0.01$), and umami ($p<0.01$) groups. However, it is interesting that this effect was reversed for the sour taste. Here, participants spent more time pumping up the balloons that ultimately exploded ($p<0.001$) (see Figure S6).

Table S 4. Average time per pump-up action in each trial, divided by taste stimuli and by Unexploded (U) / Exploded (E) balloons

	**Neutral**		**Sweet**		**Bitter**		**Sour**		**Salty**	
	U	E	U	E	U	E	U	E	U	E
M	0.34	0.32	0.44	0.29	0.28	0.24	0.32	0.45	0.29	0.34
SE	0.01	0.01	0.03	0.01	0.01	0.00	0.01	0.02	0.02	0.02

We also compared the inter-click time within the groups of unexploded and exploded balloons separately and between the six stimuli. One-way ANOVA with post-hoc Bonferroni corrections found significant differences within the groups of Unexploded and Exploded balloons ($p<0.001$). Figure S6 shows the average time it took participants to perform a click action, as well as the results of post-hoc comparisons between stimuli. Results show that (i) participants who had umami spent least time in pumping up balloons, regardless whether that balloon exploded or was cashed out; (ii) participants who had sweet spent

most time, significantly compared to other tastes ($p<0.001$), pumping up unexploded balloons; (iii) participants who had the sour taste spent significantly more time pumping up exploded balloons compared to other taste ($p<0.001$).

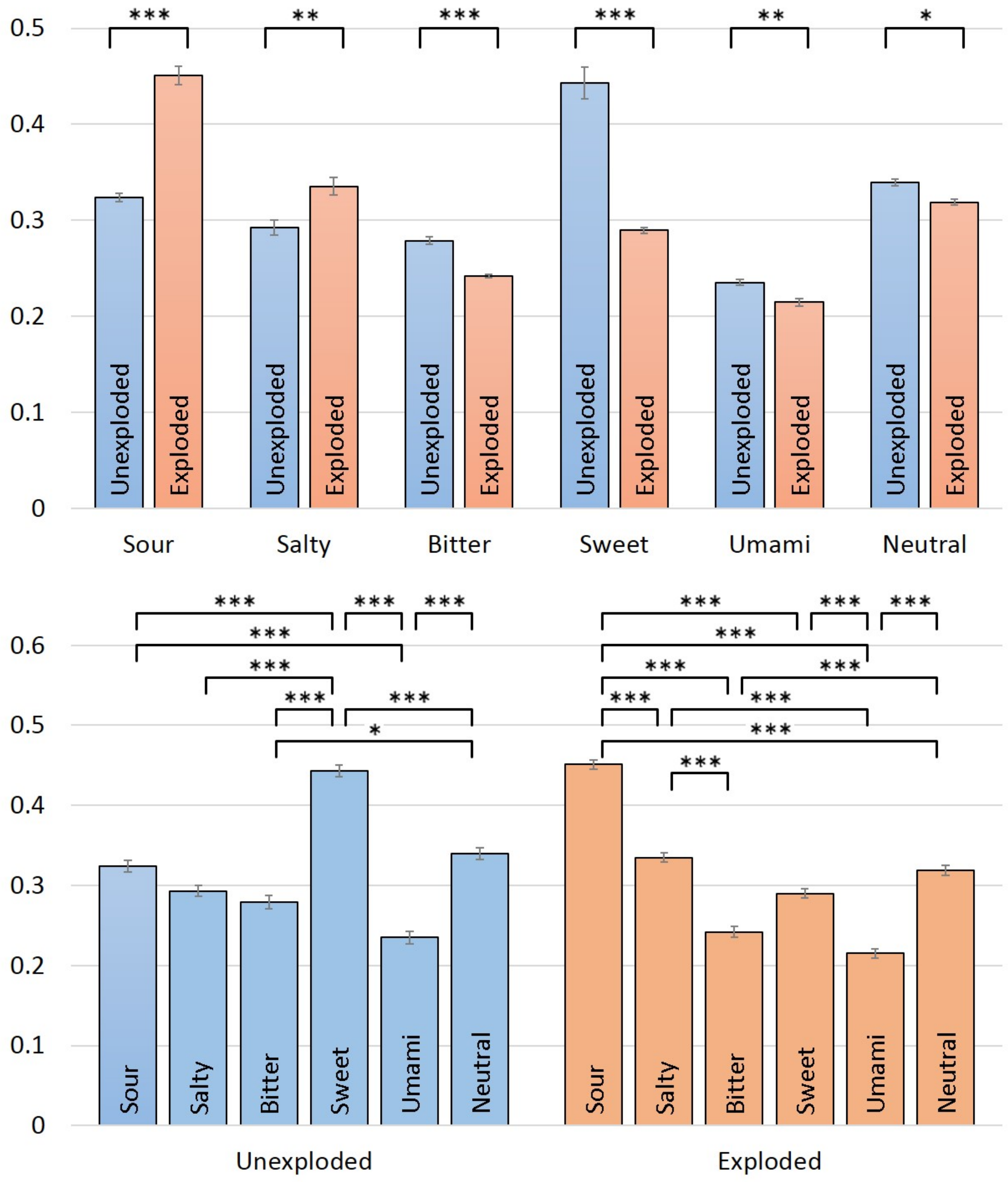

Figure S 6. Average time per pump-up action in a trial, divided by stimulus group and by Unexploded (U) / Exploded (E) balloons. Pairwise comparisons found significant differences in all stimuli between exploded and unexploded balloons (*$p<0.05$, **$p<0.01$, ***$p<0.001$). Bars represent standard error of the mean (SE).

We performed additional analysis of the average inter-click time for each trial as it progressed from trial one to trial thirty in each block. Our results showed that the average inter-click time slowly decreased over time across all taste stimuli (including the Neutral stimulus), as shown in Figure S7. We have linear regression fitting equations and R-square values for each taste group below, to show how the inter-click time slowly decreased over trials.

Sour: $y = -0.005x + 0.3801 \quad R^2 = 0.4038$

Salty: $y = -0.0054x + 0.3596 \quad R^2 = 0.4955$

Bitter: $y = -0.0073x + 0.4683 \quad R^2 = 0.4245$

Neutral: $y = -0.0062x + 0.425 \quad R^2 = 0.5077$

Umami: $y = -0.0049x + 0.3826 \quad R^2 = 0.4502$
Sweet: $y = -0.0082x + 0.5299 \quad R^2 = 0.6505$

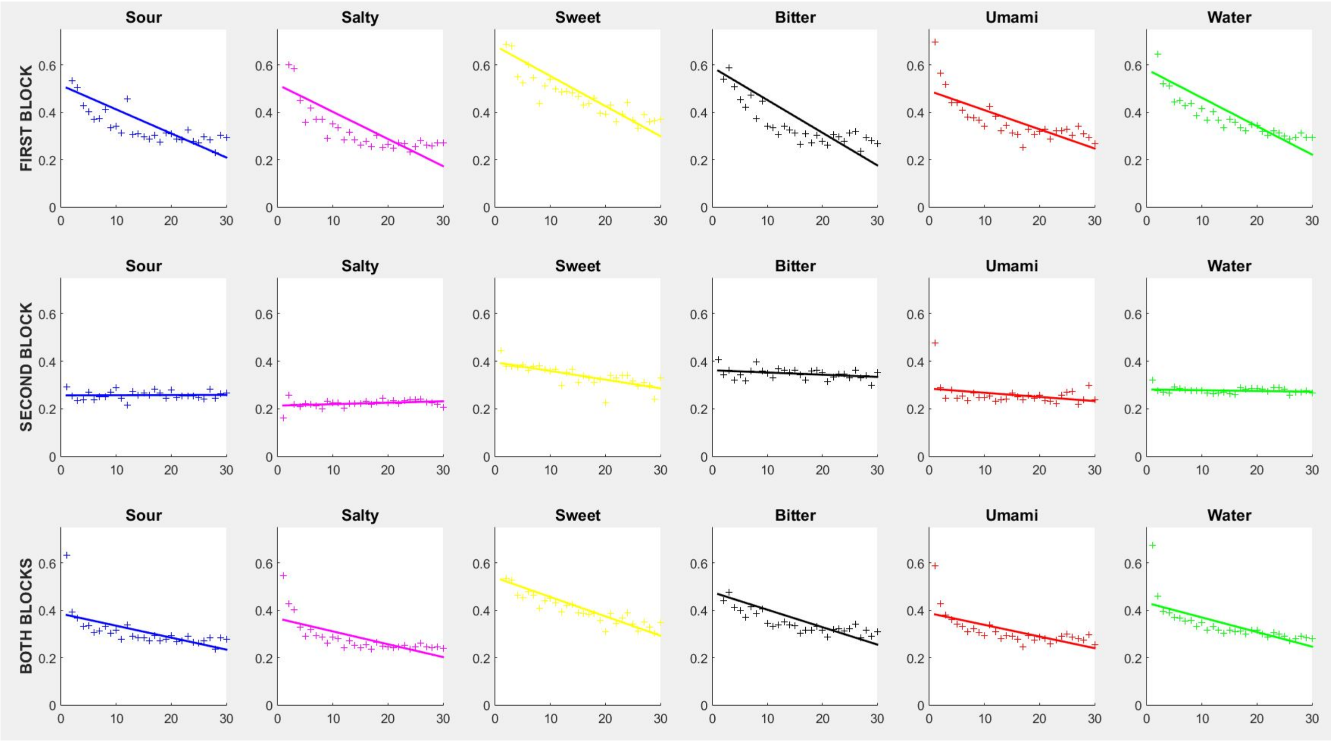

Figure S 7. Average inter-click time per trial as it progressed from trial one to trial thirty in each block, divided by stimulus group (left to right) and by block number (top to bottom: first, second, or the average of the two blocks). Solid lines represent least square linear regression fitting.

Strategies at the beginning and the end of the trial

We investigated further participants' strategies in pumping up, particularly at the beginning and at the end. To do this, we extracted the first five pumps for each stimulus group, divided by exploded and unexploded balloons. In addition, we extracted the last five pumps in the same manner to look at how participant's decision making changed at the end of the trial (either exploded or cashed out). Figure S8 and Figure S9 represent these average values of the first five (Figure S8) and last five (Figure S9) pumps. First, Figure S8 shows a clear separation between exploded and unexploded balloons from the first pump. This indicates that participants already pre-determined, from the first pump, that a balloon was going to be a risky or safe (to be cashed out) trial. It can also be seen that this effect is reversed for sour taste. Secondly, Figure S9 shows a similar comparison when participant finishing the balloon and spent more time on unexploded balloons than on exploded ones.

To analyse this effect further, we performed statistical tests with the first and the last pump separately, on the time spent on pumping up the balloons (see Figure S10). Repeated-measure ANOVA of a 6 (number of taste stimuli) x 2 (exploded, unexploded) design were performed. For the first inter-click pump, Mauchly's Test of Sphericity indicated that the assumption of shpericity had been violated $\tilde{\chi}^2(14)$= 210.98, p<0.001. Therefore, a Greenhouse-Geisser correction was used. There was a significant difference between stimuli (p<0.001) and between exploded (M 0.44 SE 0.02) vs. unexploded balloons (M 0.58 SE 0.03, p<0.001). Repeated measure ANOVA with Greenhouse-Geisser correction on the last inter-click pump indicated the same differences between stimuli (p<0.001) and between exploded (M 0.32 SE 0.01) vs. unexploded (M 0.49 SE 0.02, p<0.001).

Reward (i.e., money) gained in each group

We also calculated the money that participants earned in performing the BART task, and how it progressed over the duration of the block. Figure S11 illustrates this progression of the six taste groups. Least square linear regression shows a highly fit of the accumulating money to a linear trend. In specific, we have fitting equations and R-square values for each taste group below, in the order to show quick the money accumulated over trials. This finding is in line with the adjusted number of pump earlier.

Sour: $y = 3164x + 2579.2 \quad R^2 = 0.9916$
Salty: $y = 2912.3x + 2326.7 \quad R^2 = 0.9964$

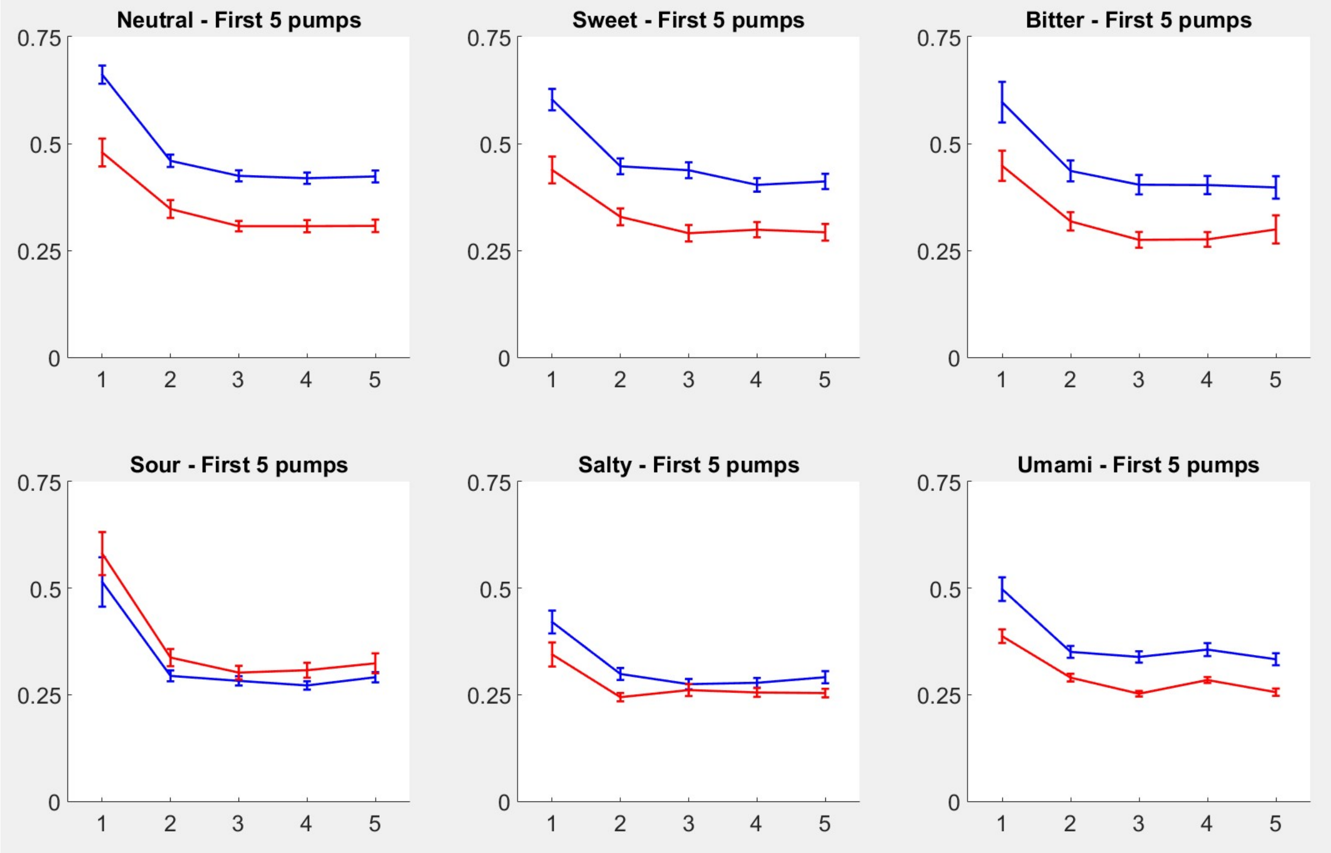

Figure S 8. Inter-click time of the first five pumps for each taste, between exploded and unexploded balloons. Bars represent standard error of the mean (SE).

Bitter: $y = 2766.6x + 156.37 \quad R^2 = 0.9875$
Neutral: $y = 2474.5x + 1449.6 \quad R^2 = 0.9891$
Umami: $y = 2133.2x + 1485.4 \quad R^2 = 0.9705$
Sweet: $y = 1958.4x + 2227.5 \quad R^2 = 0.9684$

Demographic of participants

In total, we had 70 participants in this study. Table S5 shows the demographic information for each taste group, including gender, grow up continent, age, and whether they recognized the ingested tastes.

Participants rated their preferences for each taste at the end of the experiment, after completing the BART task. Participants answered the questions "How much do you like the different tastes below?" on a scale from 1 to 6: "I don't like it at all", "I don't like it much", "Somewhat okay", "I mostly like it", "I like it very much", "I don't know". Table S6 below summarizes the mean ratings of all participants, and group by the stimulus groups (one of the five basic tastes that participants were assigned to). There was no bias of the participants' preference and the given taste.

Second Experiment - Vietnam (VN)

We collected data from 71 participants (45 females, 26 males), who were students and staffs from the local university. We collected 4,260 trials of the BART task (71 participants x 2 blocks x 30 trials). We performed an identical analysing method as in the first experiment. Specifically, we first looked at the captured SSS and BIS scores between taste groups (see Figure S12). Multivariate ANOVA with Bonferroni correction on these scores showed no significant difference between taste groups (p=0.982 for SSS, p=0.577 for BIS), and between orders (p=0.992 for SSS, p=0.749 for BIS). We also found no interaction between the order of taste with SSS scores (p=0.986), as well as with BIS scores (p=0.226). This ensured a balanced distribution of risk-taking nature within participants across all taste groups.

Among the 4,260 collected trials, we removed 1,549 trials where the balloon exploded, accounting for 36.36% of total trials. We then removed all trials where participants had the neutral stimulus in the second block, accounting for the carry-over

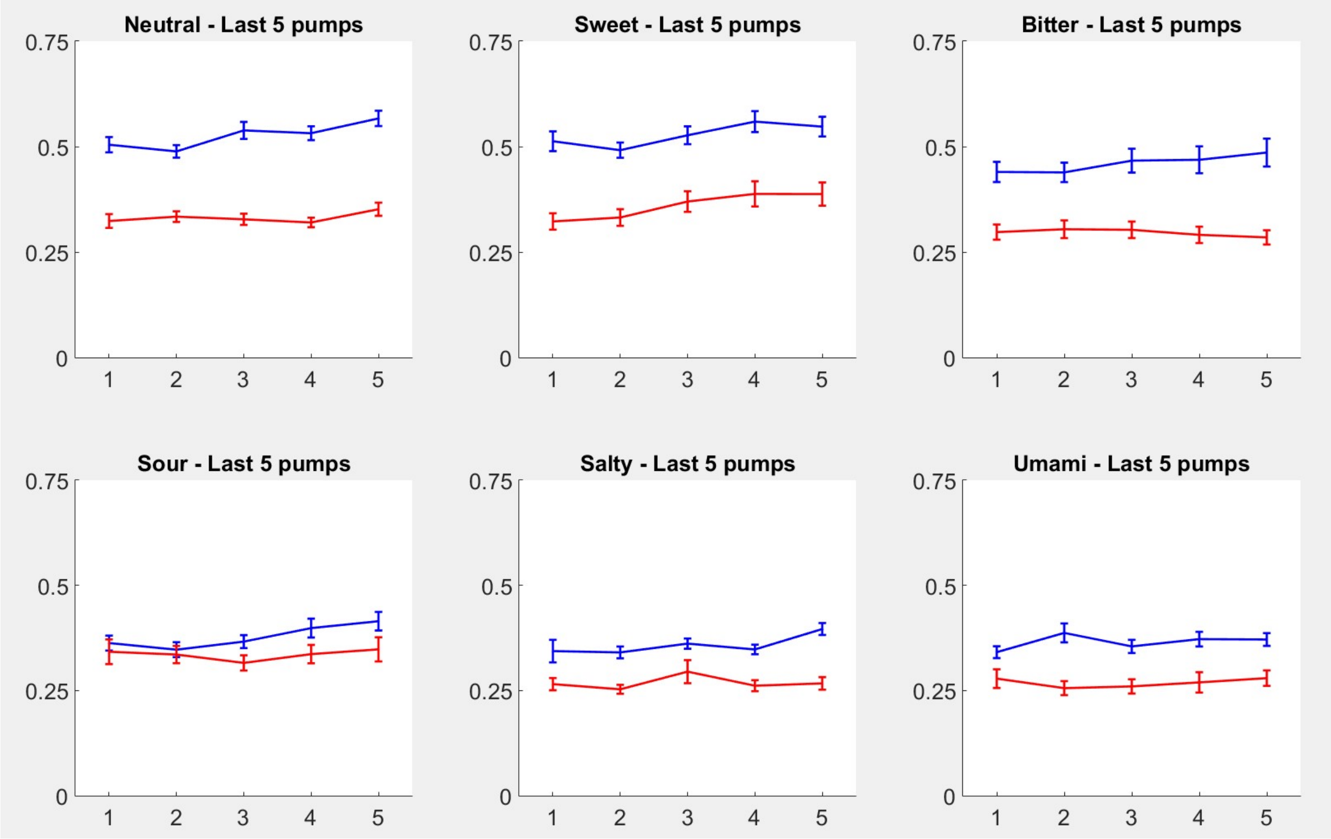

Figure S 9. Inter-click time of the last five pumps (last pump is marked #5) for each taste, between exploded and unexploded balloons. Bars represent standard error of the mean (SE).

Table S 5. Summary of participants across five basic tastes. In the experiment, participants were assigned to each taste group based on their risk-taking nature (measured by the SSS and BIS questionnaires).

**Taste/ Gender**	**Sweet**	**Bitter**	**Sour**	**Salty**	**Umami**	**Total**
Male	5	4	8	4	3	24
Female	9	10	6	10	11	46
Total	14	14	14	14	14	70
**Taste/ Grow up Continent**	**Sweet**	**Bitter**	**Sour**	**Salty**	**Umami**	**Total**
Asia	2	3	3	0	1	9
Europe	12	11	11	14	13	61
Total	14	14	14	14	14	70
**Taste/ Age**	**Sweet**	**Bitter**	**Sour**	**Salty**	**Umami**	**Total**
Mean	26.64	25.07	24.93	22.93	25.43	25.00
SD	8.50	5.79	6.65	3.43	7.71	6.41
**Taste/ Recognize**	**Sweet**	**Bitter**	**Sour**	**Salty**	**Umami**	**Total**
Yes	14	10	9	12	8	53
No	0	4	5	2	6	17
Total	14	14	14	14	14	70

effect. Following this, we removed 674 neutral trials, equivalent to 15.82% of all unexploded balloons. Figure S13 shows the percentages of the unexploded balloons for each taste group, before and after the removal of 'contaminated' balloons. We then divided the trials into 6 groups (5 tastes and 1 neutral).

For those 6 groups (5 tastes and 1 neutral), we then compared risk-taking behaviour based on the adjusted number of pumps (unexploded balloons). Using this as the dependent variable, we performed repeated measure ANOVA with Bonferoni

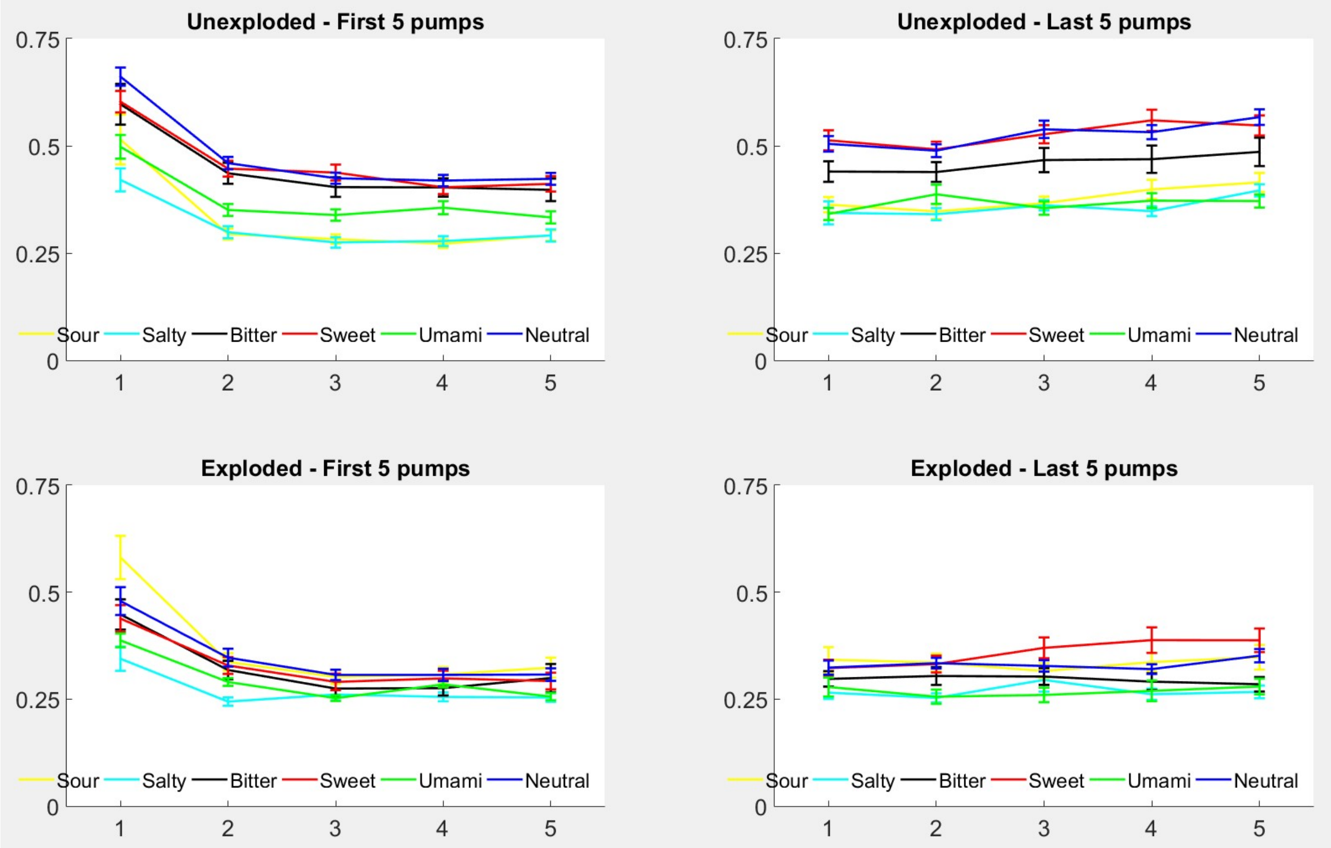

Figure S 10. Inter-click time of the first and last five pumps for each stimulus, grouped by Unexploded balloons (top) and Exploded balloons (bottom). Bars represent standard error of the mean (SE).

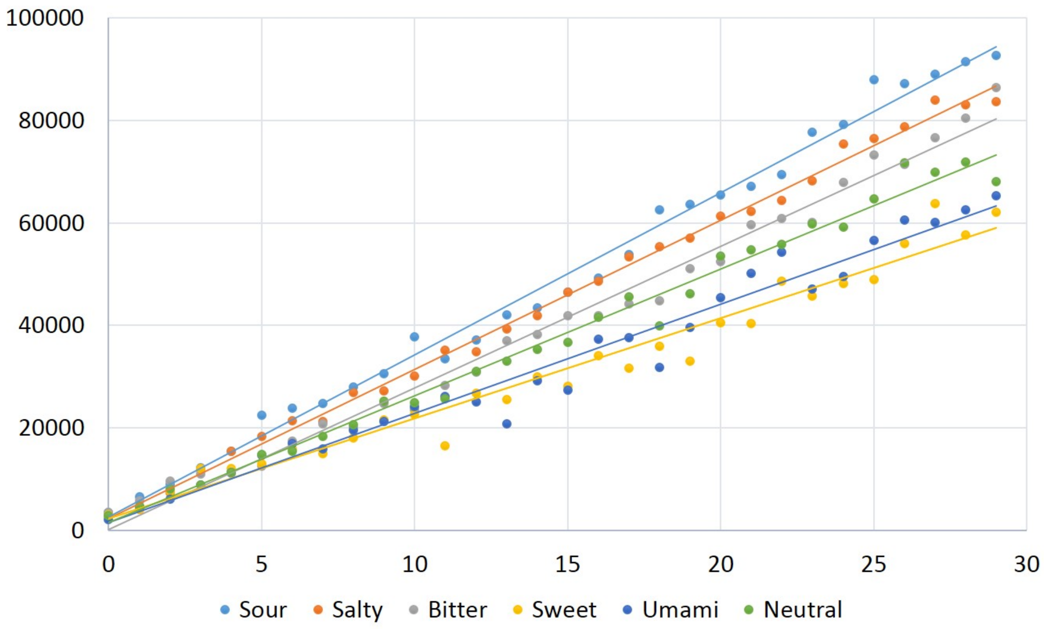

Figure S 11. Scatter plot of the average accumulate money after each trial/ balloon for each taste group, with the linear least-square fitting (vertical unit: points, horizontal unit: trial number)

Table S 6. Average ratings (± SE) of participants' preferences of each taste, categorized by the consumed stimulus group in the experiment.

Group	Sweet Rating	Sour Rating	Salty Rating	Bitter Rating	Umami Rating
Sweet	4.79 ± 0.11	3.43 ± 0.30	3.64 ± 0.26	2.50 ± 0.30	2.00 ± 0.45
Bitter	4.23 ± 0.22	3.38 ± 0.28	3.54 ± 0.34	1.77 ± 0.16	2.46 ± 0.54
Sour	4.23 ± 0.22	3.23 ± 0.25	3.62 ± 0.39	2.62 ± 0.26	2.54 ± 0.46
Salty	4.23 ± 0.38	3.00 ± 0.42	3.54 ± 0.37	2.23 ± 0.35	3.15 ± 0.53
Umami	4.62 ± 0.17	3.46 ± 0.28	3.38 ± 0.28	2.33 ± 0.22	2.54 ± 0.42

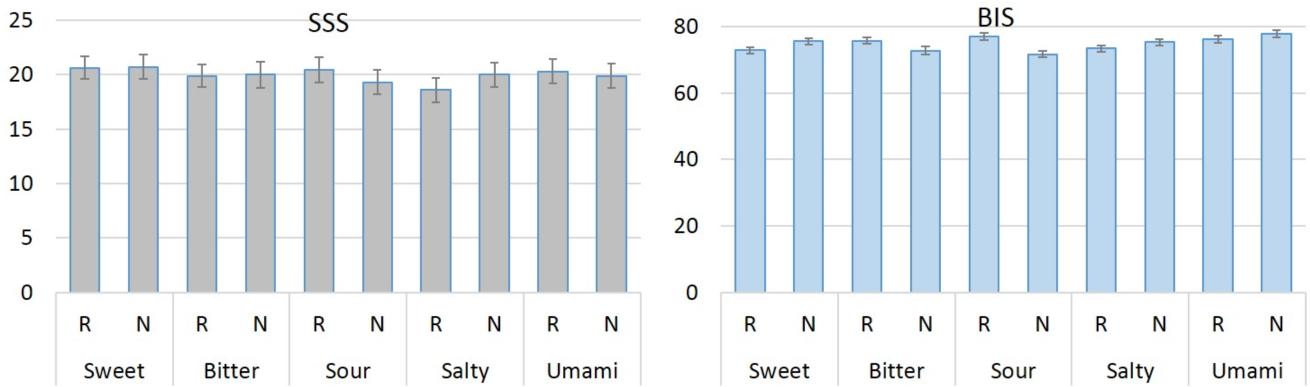

Figure S 12. Sensation Seeking Scale (SSS - top) and Barratt Impulsiveness Scale (BIS - bottom) scores of different taste groups, in two orders (R - reverse order: neutral then taste, and N - normal order: taste then neutral). Bars represent standard error of the mean (SE).

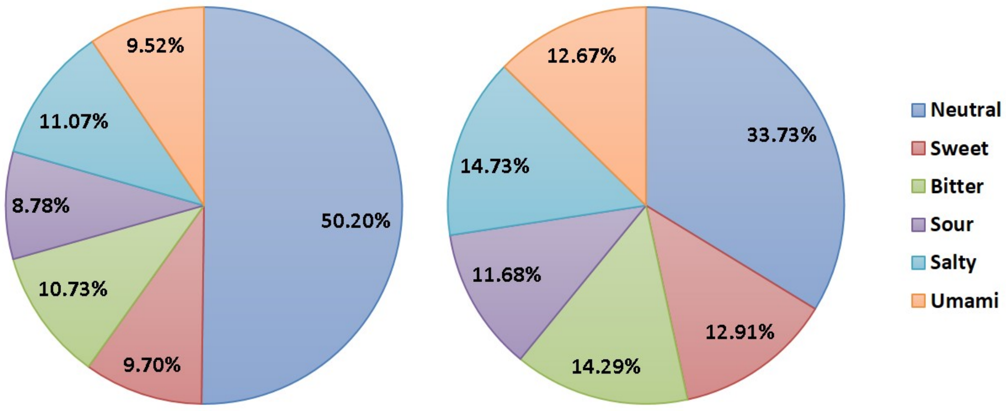

Figure S 13. Percentage of the unexploded balloons for each taste group, with the contaminated neutral stimuli (left), and uncontaminated neutral stimuli (right)

correction on each taste group comparing Neutral vs. Taste to determine the within subject effect in each group. We found the main effect of Taste in the groups of sweet ($F_{1,129}$ = 12.51, p<0.01), bitter ($F_{1,121}$ = 4.82, p<0.05), sour ($F_{1,133}$ = 5.91, p<0.05), and umami ($F_{1,143}$ = 4.05, p<0.05). We then performed an one-way ANOVA with Bonferroni correction to determine the between subject effect of the five taste groups. We found significant differences within the group ($F_{4,1345}$ = 54.18, p<0.001). Post-hoc tests show that the five groups can be arranged into an increasing order, based on their risk-taking induced effect: salty, bitter, umami, sweet, and sour. We found significant differences in all comparisons between pairs of taste groups but not between sweet vs. umami (p=0.817), bitter vs. umami (p=1.0) (see Figure S14 for illustrations of the average values and post-hoc comparisons, and Table S7 for precise summary values). In terms of gender, male participants performed significantly

higher ($p<0.05$) number of pump (M 38.34 SE 0.72) across all stimuli, comparing to female participants (M 36.05 SE 0.53).

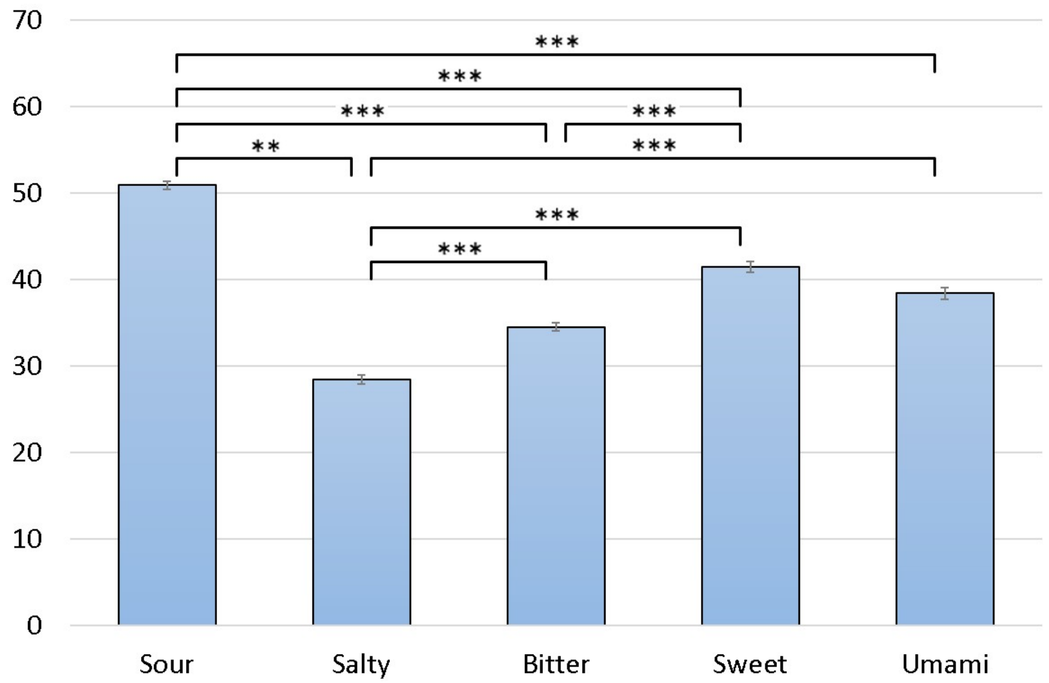

Figure S 14. Average adjusted number of pumps (the number of pumps for unexploded and uncontaminated balloons). This adjusted number of pumps is the measure of risk taking behaviour (**$p<0.01$, ***$p<0.001$). Bars represent standard error of the mean (SE).

Table S 7. Summary of adjusted number of pumps (± SE) for unexploded, uncontaminated balloons.

	Sweet	Bitter	Sour	Salty	Umami
Reverse	39.42 ± 1.85	36.56 ± 1.22	49.85 ± 1.19	27.98 ± 1.42	38.03 ± 1.64
Normal	43.12 ± 1.70	35.63 ± 1.42	51.98 ± 1.60	28.95 ± 1.51	38.75 ± 2.15
All balloons	41.47 ± 1.25	36.00 ± 0.98	50.88 ± 0.99	28.46 ± 1.03	38.37 ± 1.33

Influence of the stimuli's pleasantness

To investigate the influence of the stimuli's pleasantness on risk-taking behaviour, we calculated the correlation between the rating of stimulus pleasantness (given by participants right after ingesting the stimulus) and the adjusted number of pumps for that stimulus' block. The Pearson correlation test also shows no correlation between the pleasantness ratings of the stimuli and the adjusted number of pumps ($r=0.13$, $p=0.19$). Specific for each individual stimulus, we also found no correlation in neutral ($r=0.12$, $p=0.49$), sweet ($r=0.32$, $p=0.24$), bitter ($r=0.36$, $p=0.21$), sour ($r=-0.02$, $p=0.95$), salty ($r=-0.40$, $p=0.16$), and umami ($r=0.34$, $p=0.23$). Participants rated sweet slightly pleasant (mean 0.47 ± 0.26), bitter slightly unpleasant (-0.24 ± 0.32), sour as neither pleasant nor unpleasant (0.0 ± 0.34), salty slightly unpleasant (-0.36 ± 0.26), umami neither pleasant nor unpleasant (0.00 ± 0.30), and neutral slightly pleasant (0.85 ± 0.17).

Participant behaviour over time

To investigate the temporal pattern of participants' performance in the six groups, we analysed clicking behaviour in the BART task (the time elapsed between the current pump and the previous pump). First, we aligned all trials with the first click to pump up the balloon. We then averaged all trials of all participants for each taste, and for exploded/unexploded balloons separately. We then performed repeated measure ANOVA with Bonferoni correction on each taste group comparing Neutral vs. Taste to determine the within subject effect in each taste group. We found the main effect of Taste in the groups of sweet ($F_{1,103} = 14.82$, $p<0.001$), bitter ($F_{1,74} = 35.01$, $p<0.001$), sour ($F_{1,92} = 29.72$, $p<0.001$), and umami ($F_{1,85} = 67.63$, $p<0.001$). We then

performed an one-way ANOVA with Bonferroni correction to determine the between subject effect of the five taste groups. We found significant differences within the group ($F_{4,453} = 2.82$, $p<0.05$). Post-hoc comparisons show significant difference in pairwise comparisons of (sour vs. sweet) with $p<0.05$ but not in other pairs. Figure S15 illustrates the average inter-click time for a pump-up action of both exploded and unexploded balloons, divided by the six stimuli, and Table S8 shows the precise values.

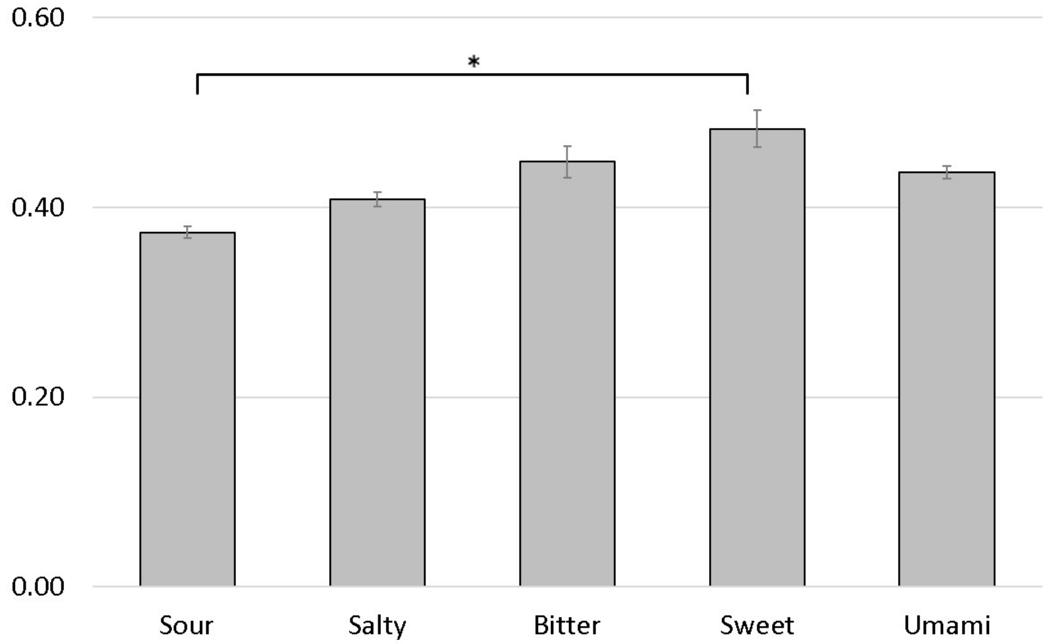

Figure S 15. The average inter-click time between pump-up actions for uncontaminated balloons, divided into six stimulus groups (*$p<0.05$). Bars represent standard error of the mean (SE).

Table S 8. Average time participants spent pumping up balloons, divided by different stimuli (5 basic tastes and 1 neutral).

	Sweet	Bitter	Sour	Salty	Umami
M	0.48	0.45	0.37	0.41	0.44
SE	0.04	0.03	0.01	0.01	0.01

In addition, we looked into details of exploded and unexploded balloons separately. Figure S16 and Table S9 show the average time spent for a pump-up action, averagely over all trials of each stimulus, and separately between exploded and unexploded balloons. Pair-wise comparisons showed significant difference between them in all stimuli except sweet. Specifically, participants spent significantly more time pumping up balloons that were going to be cashed out than the ones that were going to explode in the neutral ($p<0.001$), bitter ($p<0.001$), sour ($p<0.001$), salty ($p<0.001$), and umami ($p<0.01$) groups. However, we could not observe this effect in sweet taste ($p>0.05$) (see Figure S16).

We also compared the click inter-click time within the groups of unexploded and exploded balloons separately and between the six stimuli. One-way ANOVA with post-hoc Bonferroni corrections found significant differences within the groups of Unexploded and Exploded balloons ($p<0.001$). Figure S16 shows the average time it took participants to perform a click action, as well as the results of post-hoc comparisons between stimuli.

We performed additional analysis of the average inter-click time for each trial as it progressed from trial one to trial thirty in each block. Our results showed that the average inter-click time slowly decreased over time across all taste stimuli (including the Neutral stimulus), as shown in Figure S17. We have linear regression fitting equations and R-square values for each taste group below, to show how the inter-click time slowly decreased over trials.

Sour: $y = -0.0042x + 0.3962 \quad R^2 = 0.5069$

Salty: $y = -0.0069x + 0.589 \quad R^2 = 0.5750$

Bitter: $y = -0.0051x + 0.4653 \quad R^2 = 0.7020$

Neutral: $y = -0.0074x + 0.5261 \quad R^2 = 0.8053$

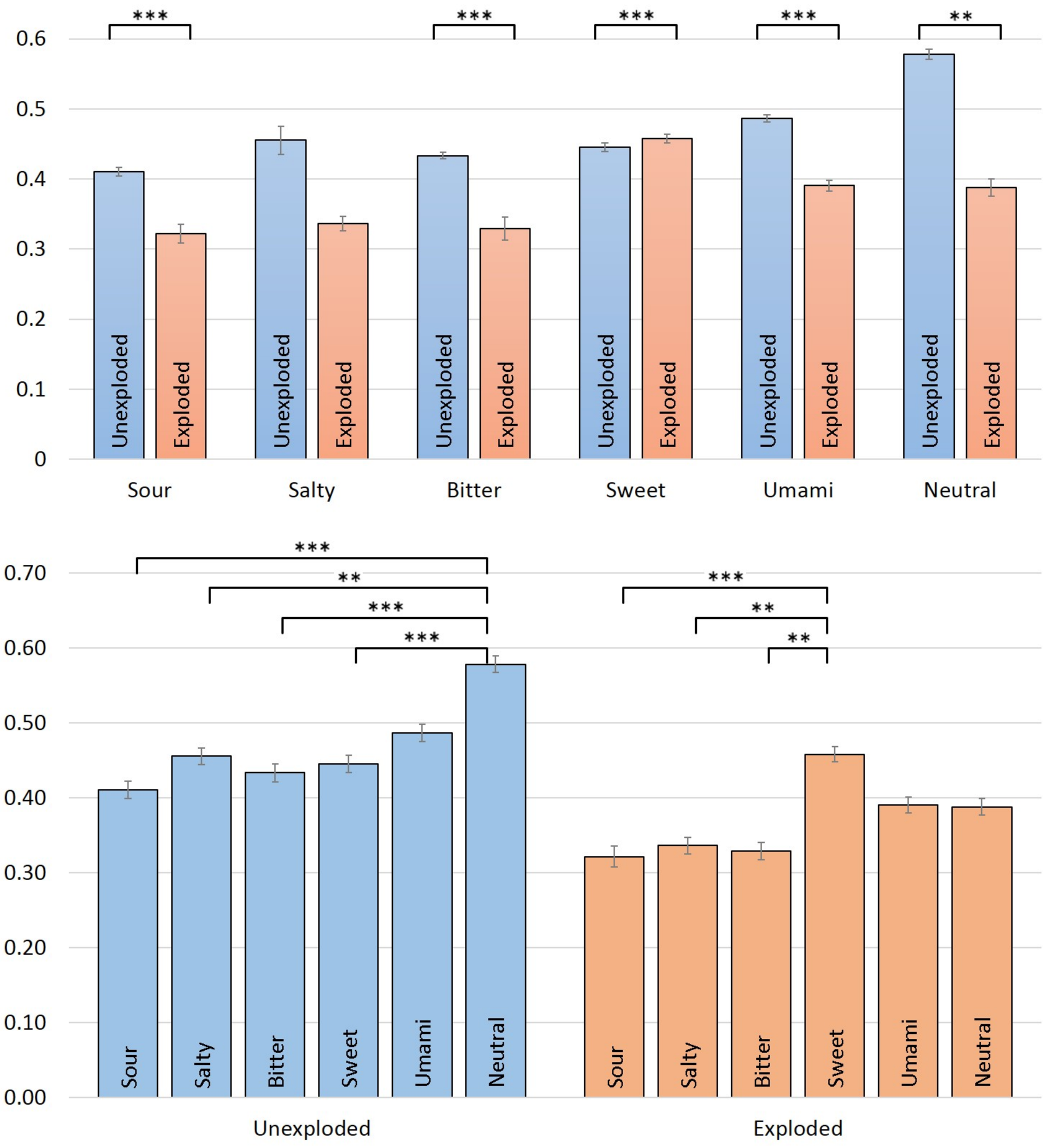

Figure S 16. The average inter-click time between pump-up actions for uncontaminated balloons, divided into six stimulus groups (*p<0.05). Bars represent standard error of the mean (SE).

Umami: $y = -0.0079x + 0.5043 \quad R^2 = 0.7879$
Sweet: $y = -0.0041x + 0.4537 \quad R^2 = 0.4091$

Strategies at the beginning and the end of the trial

We investigated further participants' strategies in pumping up, particularly at the beginning and at the end. To do this, we extracted the first five pumps for each stimulus group, divided by exploded and unexploded balloons. In addition, we extracted the last five pumps in the same manner to look at how participant's decision making changed at the end of the trial (either exploded or cashed out). Figure S18 and Figure S19 represent these average values of the first five (Figure S18) and last five (Figure S19) pumps. First, Figure S18 shows a clear separation between exploded and unexploded balloons from the first pump. This indicates that participants already pre-determined, from the first pump, that a balloon was going to be a risky or safe (to be cashed out) trial. It can also be seen that this effect is reversed for sour taste. Secondly, Figure S19 shows a similar comparison when participant finishing the balloon and spent more time on unexploded balloons than on exploded ones.

To analyse this effect further, we performed statistical tests with the first and the last pump separately, on the time spent on pumping up the balloons. Repeated-measure ANOVA of a 6 (number of taste stimuli) x 2 (exploded, unexploded) design were

Table S 9. Average time per pump-up action in each trial, divided by stimulus group and by Unexploded (U) / Exploded (E) balloons

	Neutral		Sweet		Bitter	
	U	E	U	E	U	E
M	0.58	0.39	0.45	0.46	0.43	0.33
SE	0.03	0.01	0.02	0.04	0.03	0.01

	Sour		Salty		Umami	
	U	E	U	E	U	E
M	0.41	0.32	0.46	0.34	0.49	0.39
SE	0.01	0.01	0.02	0.01	0.02	0.01

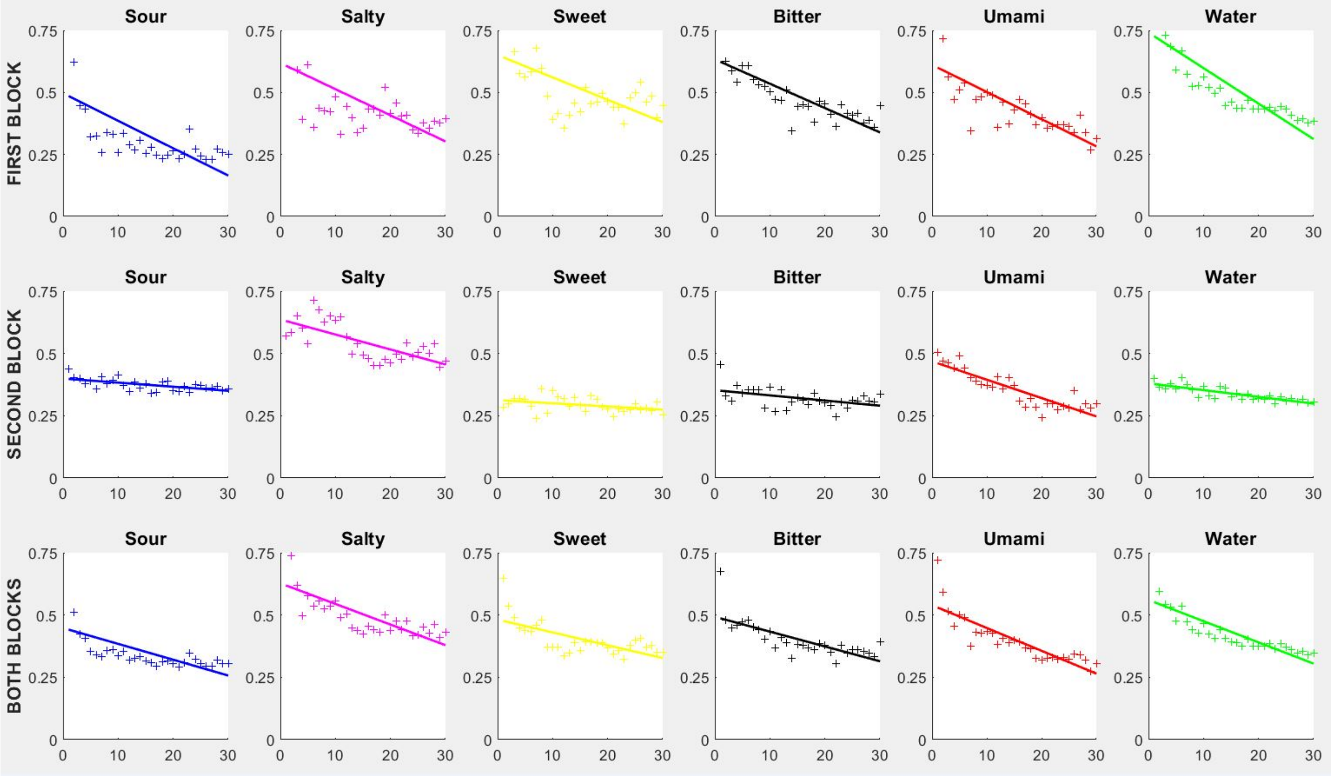

Figure S 17. Average inter-click time per trial in a block, divided by stimulus group (left to right) and by block number (top to bottom: first, second, or the average of the two blocks). Solid lines represent least square linear regression fitting.

performed. For the first inter-click pump, Mauchly's Test of Sphericity indicated that the assumption of sphericity had been violated $\tilde{\chi}^2(14)$= 33.73, p<0.01. Therefore, a Greenhouse-Geisser correction was used. There was a significant difference between stimuli (p<0.001) but not between exploded (M 0.62 SE 0.03) vs. unexploded balloons (M 0.68 SE 0.03, p=0.11). Repeated measure ANOVA with Greenhouse-Geisser correction on the last inter-click pump indicated a significant effect between stimuli (p<0.001) and between exploded (M 0.44 SE 0.02) vs. unexploded balloons (M 0.62 SE 0.02, p<0.001).

Reward (i.e., money) gained in each group

We also calculated the money that participants earned in performing the BART task, and how it progressed over the duration of the block. Figure S21 illustrates this progression of the six taste groups. Least square linear regression shows a highly fit of the accumulating money to a linear trend. In specific, we have fitting equations and R-square values for each taste group below, in the order of how quick the money accumulated over trials. This finding is in line with the adjusted number of pump earlier.

Sour: $y = 3656.3x + 1740.9 \quad R^2 = 0.9976$

Neutral: $y = 3200.6x + 587.86 \quad R^2 = 0.9991$

Bitter: $y = 3076.3x + 1008.8 \quad R^2 = 0.9984$

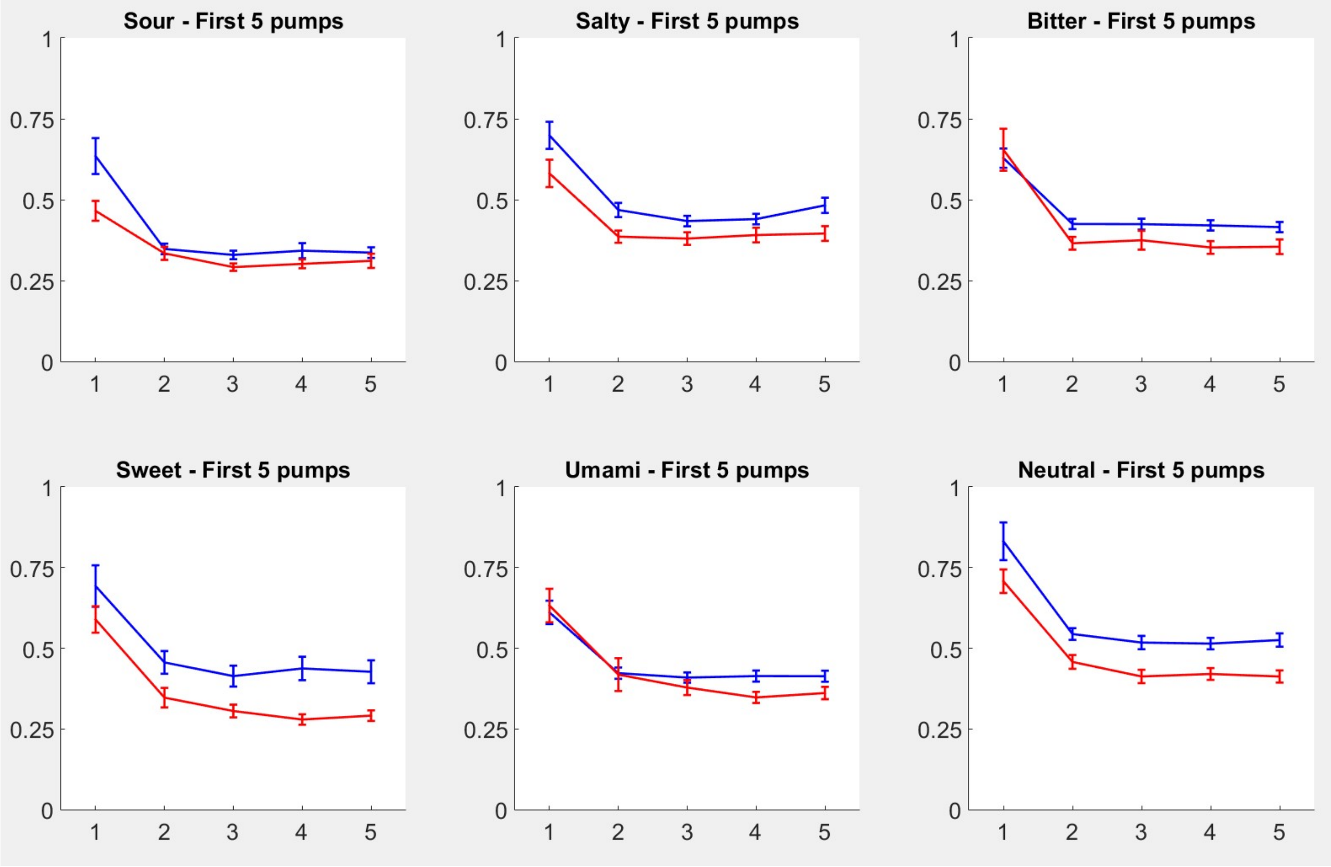

Figure S 18. Inter-click time of the first five pumps for each taste, between exploded and unexploded balloons. Bars represent standard error of the mean (SE).

Sweet: $y = 3100.1x + 984.71 \quad R^2 = 0.9985$
Salty: $y = 2543.6x + 2610 \quad R^2 = 0.9988$
Umami: $y = 2947.9x + 4269.9 \quad R^2 = 0.9973$

Demographic of participants

In total, we had 71 participants in this study. Table S10 shows the demographic information for each taste group, including gender, grow up continent, age, and whether they recognized the ingested tastes.

Participants rated their preferences for each taste at the end of the experiment, after completing the BART task. Participants answered the questions "How much do you like the different tastes below?" on a scale from 1 to 6: "I don't like it at all", "I don't like it much", "Somewhat okay", "I mostly like it", "I like it very much", "I don't know". Table S11 below summarizes the mean ratings of all participants, and group by the stimulus groups (one of the five basic tastes that participants were assigned to). There was no bias of the participants' preference and the given taste.

Interestingly, we found that the five participants, who did not recognize umami taste, indicated that they had salty taste (four participants) and something between sweet and sour (1 participant). Additionally, the two participants who did not recognize salty taste, indicated that they had umami taste.

Participants rated their preferences for each taste at the end of the experiment, after completing the BART task. Participants answered the questions "How much do you like the different tastes below?" on a scale from 1 to 6: "I don't like it at all", "I don't like it much", "Somewhat okay", "I mostly like it", "I like it very much", "I don't know". Table 11 below summarizes the mean ratings of all participants, and group by the stimulus groups (one of the five basic tastes that participants were assigned to). There was no bias of the participants' preference and the given taste.

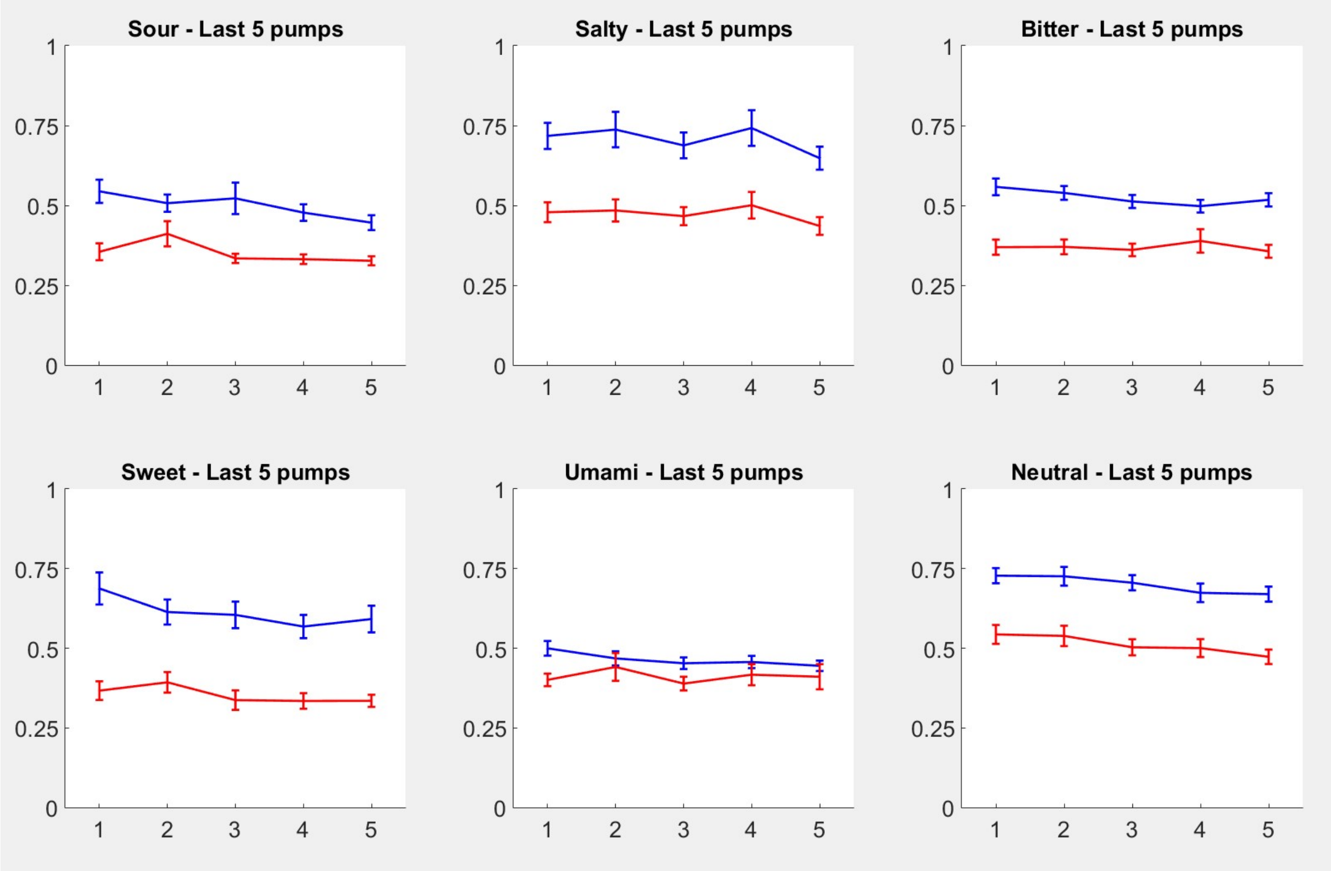

Figure S 19. Inter-click time of the last five pumps (last pump is marked #1) for each taste, between exploded and unexploded balloons. Bars represent standard error of the mean (SE).

Third experiment – UK

We collected data from 27 participants (22 females, 5 males), who were students and staffs from the local university. We collected 1,620 trials of the BART task (27 participants x 2 blocks x 30 trials).

Similar to the previous two experiments, we first looked at the captured SSS and BIS scores between taste groups, their orders, and Cognitive Reflection Test levels (CRT - low or high). Multivariate ANOVA with Bonferroni correction on these scores showed no significant difference between order (p=0.40 for SSS, p=0.07 for BIS), and two levels of cognitive ability (p=0.13 for SSS, p=0.07 for BIS). This ensured a balanced distribution of risk-taking nature within participants across all order and cognitive ability levels (see Figure S22).

Among the 1,620 collected trials, we only considered those where the balloon did not explode (participants chose to collect the current points instead of to continue pumping). Thus, we removed 610 trials where the balloon exploded, accounting for 37.65% of total trials. Because each participant performed two blocks of the BART task, one block with sour taste and another with the neutral stimuli, we separated the trials into two groups of the sour taste and the neutral stimulus. Following the same analysing method for the UK and VN datasets, we removed trials of neutral stimulus taken in the second block (the contaminated trials). Following this, we removed 229 trials, equivalent to 22.67% of all unexploded balloons.

We then performed multivariate ANOVA with Bonferroni correction with:

- Independent variables
  - 2 tastes: sour and neutral
  - 2 levels based on the number of correct answer of the CRT test: Intuitive (0 or 1 correct answer) and Analytic (2 or 3 correct answer).
- Dependent variable: adjusted number of pumps for unexploded and uncontaminated balloons.

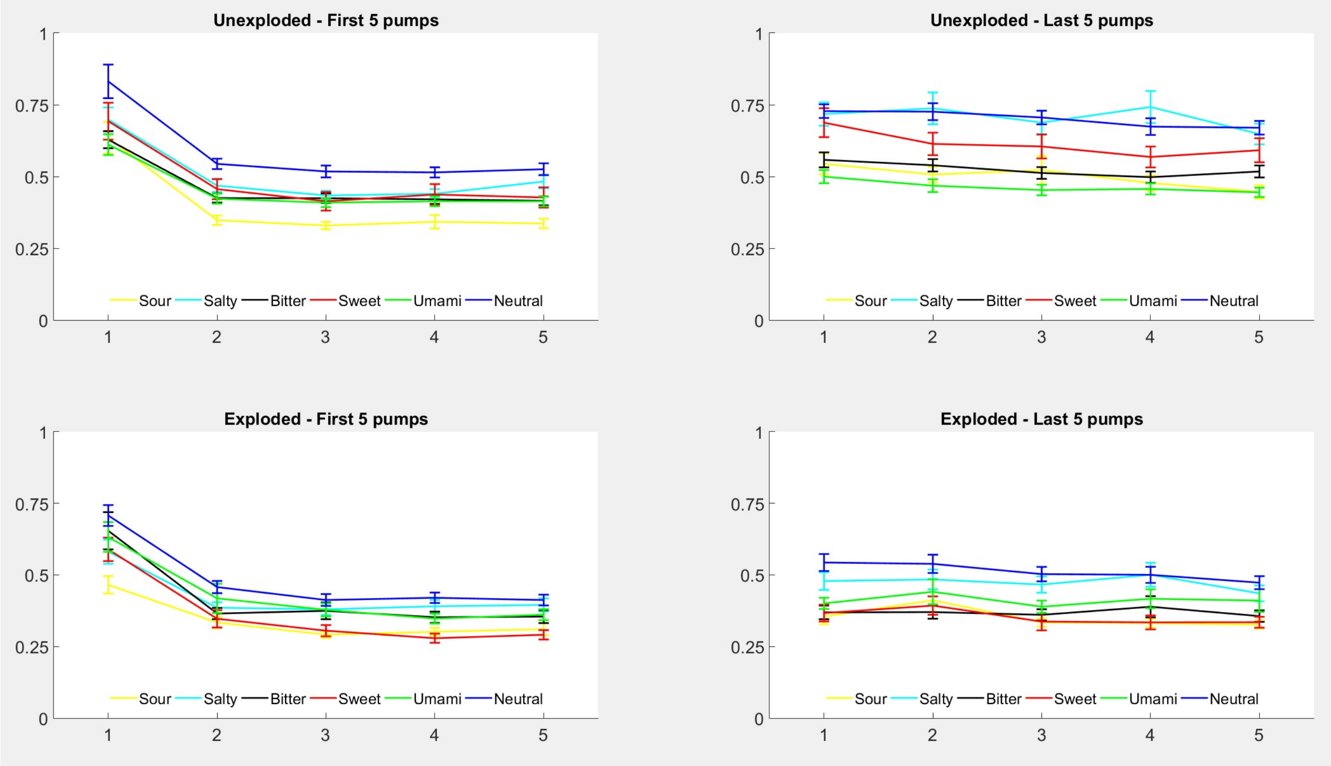

Figure S 20. Inter-click time of the first and last five pumps for each stimulus, grouped by Unexploded balloons (top) and Exploded balloons (bottom). Bars represent standard error of the mean (SE).

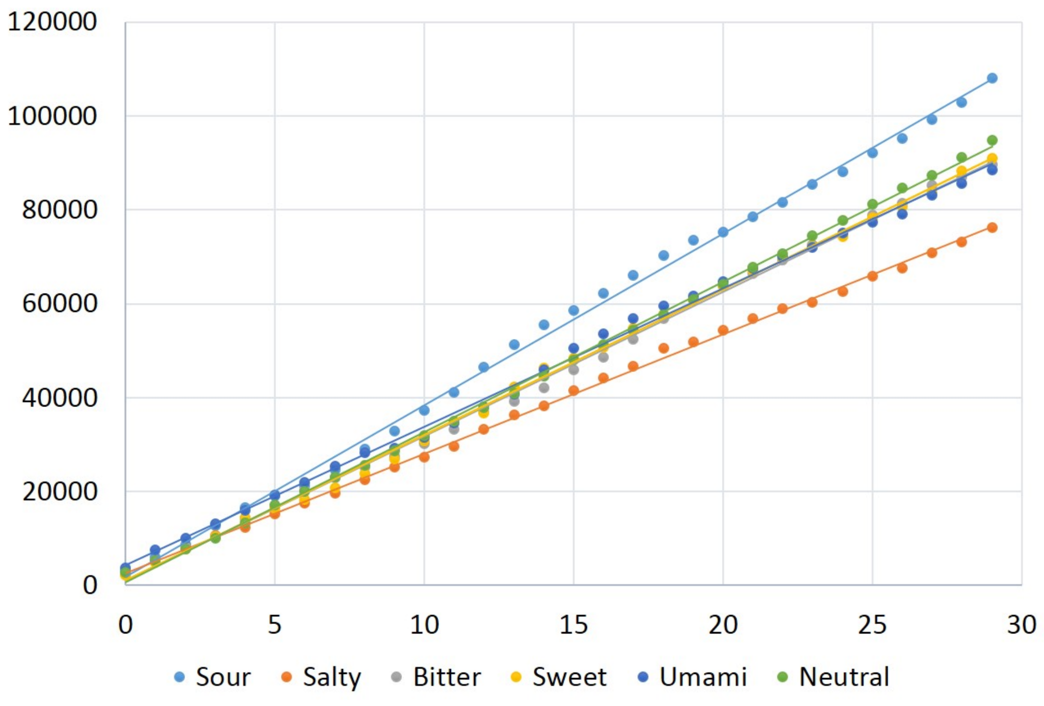

Figure S 21. Scatter plot of the average accumulate money after each trial/ balloon for each taste group, with the linear least-square fitting (vertical unit: points, horizontal unit: trial number).

Table S 10. Summary of participants across five basic tastes. In the experiment, participants were assigned to each taste group based on their risk-taking nature (measured by the SSS and BIS questionnaires).

Taste/ Gender	Sweet	Bitter	Sour	Salty	Umami	Total
Male	4	5	5	4	8	26
Female	11	9	9	10	6	45
Total	15	14	14	14	14	71
**Taste/ Age**	**Sweet**	**Bitter**	**Sour**	**Salty**	**Umami**	**Total**
Mean	20.20	19.50	20.15	22.14	22.00	20.20
SD	1.72	1.12	1.35	3.94	4.61	1.72
**Taste/ Recognize**	**Sweet**	**Bitter**	**Sour**	**Salty**	**Umami**	**Total**
Yes	14	12	14	12	9	61
No	1	2	0	2	5	10
Total	15	14	14	14	14	71

Table S 11. Average ratings (± SE) of participants' preferences of each taste, categorized by the consumed stimulus group in the experiment.

Group	Sweet Rating	Sour Rating	Salty Rating	Bitter Rating	Umami Rating
Sweet	3.8 ± 0.32	3.47 ± 0.28	2.86 ± 0.22	2.40 ± 0.31	2.54 ±0.35
Bitter	4.08 ± 0.83	3.31 ± 0.99	3.00 ± 0.78	2.15 ± 1.17	2.46 ± 1.34
Sour	4.14 ± 1.06	3.29 ± 1.22	3.14 ± 0.83	2.29 ± 1.16	2.15 ± 1.1
Salty	4.36 ± 0.72	3.43 ± 0.98	3.21 ± 0.56	2.07 ± 0.96	2.31 ± 1.2
Umami	4.36 ± 0.61	3.07 ± 1.16	3.14 ± 0.74	2.00 ± 1.18	2.14 ± 0.83

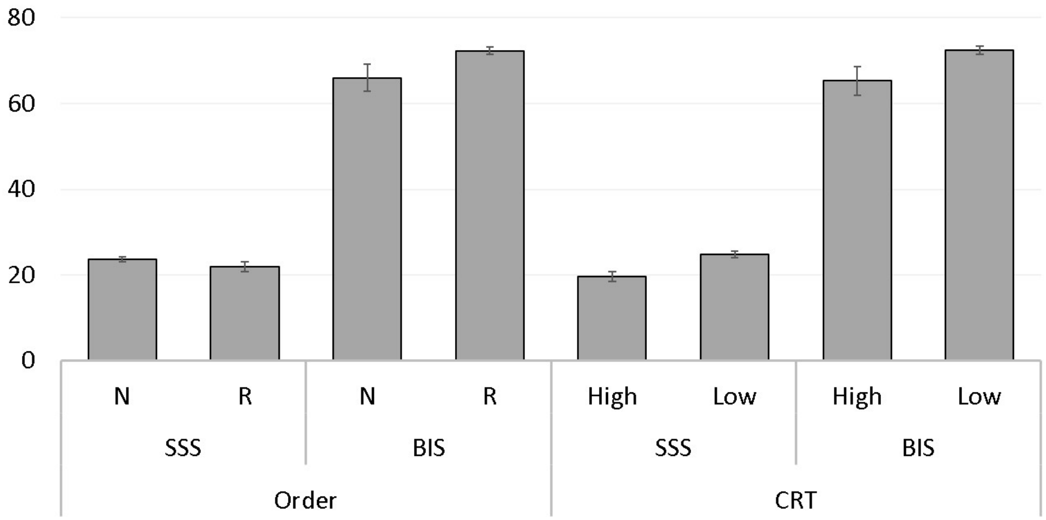

Figure S 22. Sensation Seeking Scale (SSS) and Barratt Impulsiveness Scale (BIS) scores of (left) two orders (R - reverse order: neutral then sour taste, and N - normal order: sour taste then neutral); and (right) two levels of cognitive ability (Low – CRT correct answers of 0 or 1, High – CRT correct answers of 2 or 3). Bars represent standard error of the mean (SE).

We found a significant difference in the adjusted number of pump between the two taste groups ($p<0.05$) as well as between two levels of intuitive/ analytic ($p<0.05$). Additionally, pairwise comparisons found significant differences between intuitive vs. analytic of each taste ($p<0.05$). Figure S23 illustrates these values and comparisons and Table 12 for precise values.

Influence of the stimuli's pleasantness

To investigate the influence of the stimuli's pleasantness on risk-taking behaviour, we calculated the correlation between the rating of stimulus pleasantness (given by participants right after ingesting the stimulus) and the adjusted number of pumps for that stimulus' block. The Pearson correlation test found correlation between the pleasantness ratings of the stimuli and the

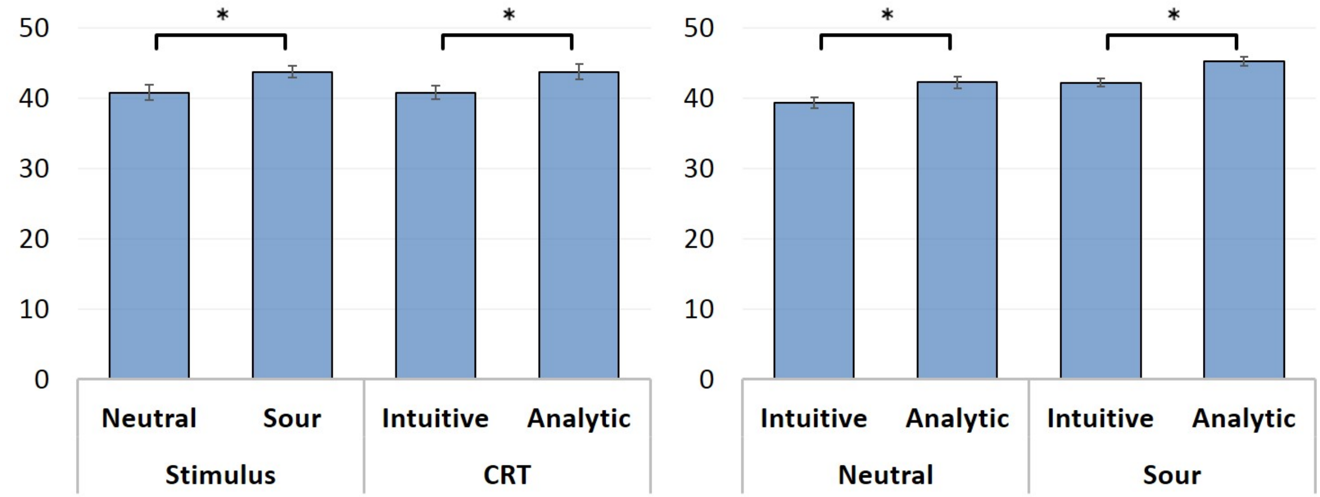

Figure S 23. Average adjusted number of pumps (the number of pumps for unexploded and uncontaminated balloons) for each taste with two cognitive ability levels, as well as for tastes and CRT levels separately. This adjusted number of pumps is the measure of risk taking behaviour (*p<0.05). Bars represent standard error of the mean (SE).

Table S 12. Summary of adjusted number of pumps for unexploded, uncontaminated balloons.

**Neutral**	Intuitive	M 39.37	SE 1.52
	Analytic	M 42.27	SE 1.66
**Sour**	Intuitive	M 42.24	SE 1.08
	Analytic	M 45.27	SE 1.30
**Taste**	Neutral	M 40.82	SE 1.13
	Sour	M 43.76	SE 0.85
**CRT**	Intuitive	M 40.80	SE 0.93
	Analytic	M 43.77	SE 1.05

adjusted number of pumps ($r=-0.37$, $p<0.05$). Specific for each individual stimulus, we also found no correlation in neutral ($r=-0.25$, $p=0.41$) but significant correlation in sour ($r=-0.42$, $p<0.05$). Participants rated sour as slightly unpleasant (-0.36 ± 0.24) and neutral slightly pleasant (0.46 ± 0.18).

Participant behaviour over time

To investigate the temporal pattern of participants' performance in the two groups, we analysed clicking behaviour in the BART task (the time elapsed between the current pump and the previous pump). First, we aligned all trials with the first click to pump up the balloon. We then averaged all trials of all participants for each taste, and for exploded/unexploded balloons separately. Figure S24 illustrates the average inter-click time for a pump-up action of both exploded and unexploded balloons, divided by the sour and neutral stimuli, and Table S13 shows the precise values. Independent t-test found no significant difference between the two stimuli.

Table S 13. Average time participants spent pumping up balloons (the inter-click time), divided by two stimuli.

	**Neutral**	**Sour**
Average	0.33	0.31
SD	0.17	0.16

In addition, we looked into details of exploded and unexploded balloons separately. Figure S25 and Table S14 show the average time spent for a pump-up action, averagely over all trials of each stimulus, and separately between exploded and unexploded balloons. Pair-wise comparisons found no significant difference between all pairs of comparisons.

We performed additional analysis of the average inter-click time for each trial as it progressed from trial one to trial thirty in each block. Our results showed that the average inter-click time slowly decreased over time across all taste stimuli (including

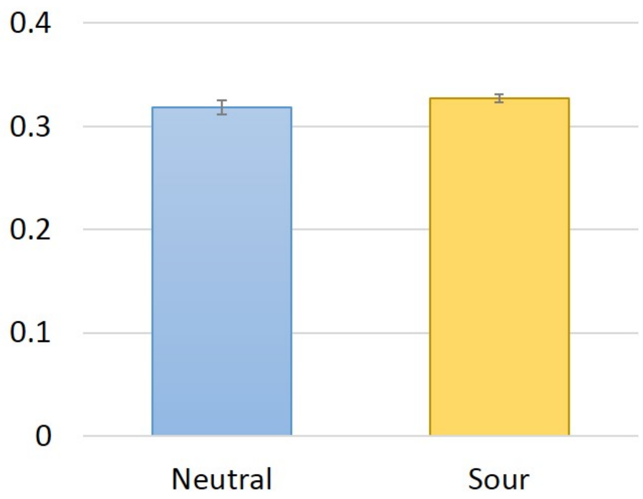

Figure S 24. The average inter-click time between pump-up actions for uncontaminated balloons for both exploded and unexploded balloons. Bars represent standard error of the mean (SE).

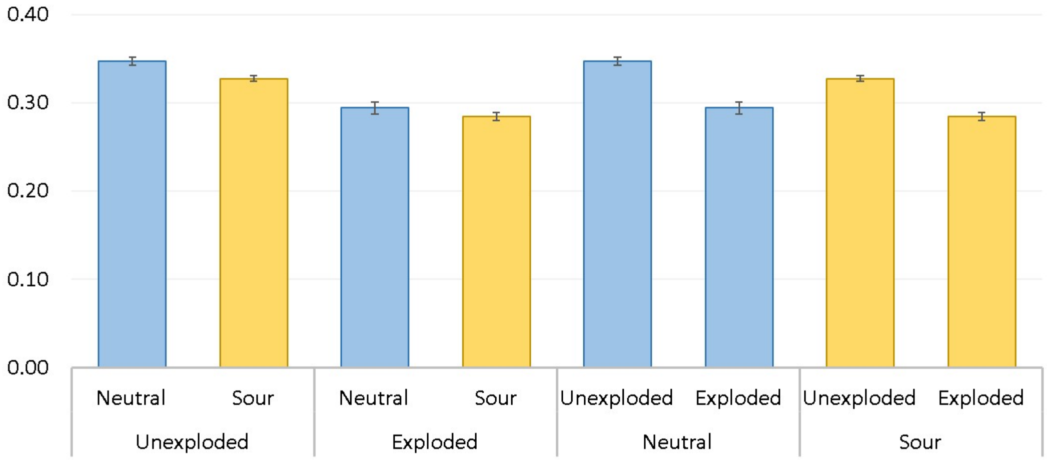

Figure S 25. Average time per pump-up action in a trial, divided by stimulus group and by Unexploded (U) / Exploded (E) balloons. Bars represent standard error of the mean (SE).

Table S 14. Average time per pump-up action in each trial, divided by stimulus group and by Unexploded (U) / Exploded (E) balloons.

	**Neutral**		**Sour**	
	U	E	U	E
Mean	0.35	0.29	0.33	0.28
SE	0.00	0.01	0.00	0.00

the Neutral stimulus), as shown in Figure S26. We have linear regression fitting equations and R-square values for each taste group below, to show how the inter-click time slowly decreased over trials.

Sour: $y = -0.0039x + 0.3625$ $R^2 = 0.7297$

Neutral: $y = -0.0034x + 0.3406$ $R^2 = 0.5782$

Strategies at the beginning and the end of the trial

We investigated further participants' strategies in pumping up, particularly at the beginning and at the end. To do this, we extracted the first five pumps for sour and neutral group, divided by exploded and unexploded balloons. In addition, we

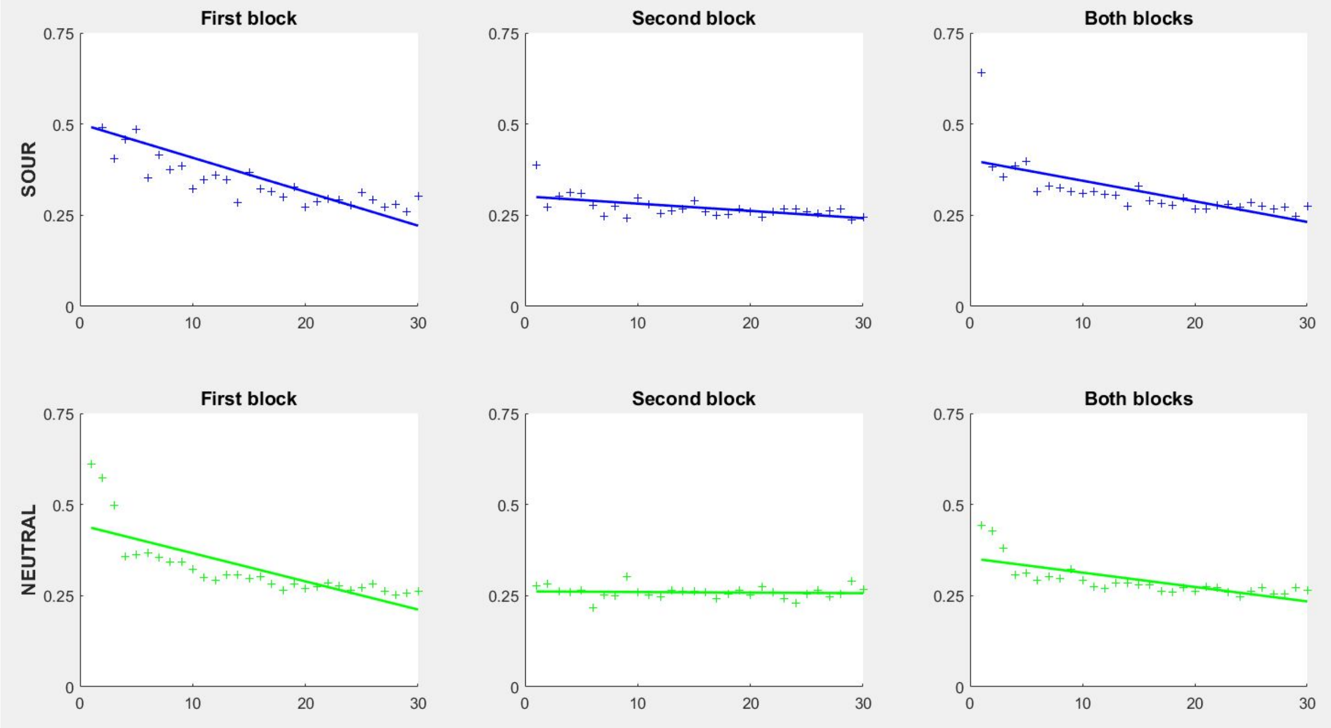

Figure S 26. Average inter-click time per trial as it progressed from trial one to trial thirty in each block, divided by stimulus group (sour - top and Neutral - bottom) and by block number (left to right: first, second, or the average of the two blocks). Solid lines represent least square linear regression fitting.

extracted the last five pumps in the same manner to look at how participant's decision making changed at the end of the trial (either exploded or cashed out). Figure S27 and Figure S28 represents these average values of the first five and last five pumps, respectively.

Reward (i.e., money) gained in each group

We also calculated the money that participants earned in performing the BART task, and how it progressed over the duration of the block. Figure S29 illustrates this progression of the sour taste and neutral group. Least square linear regression shows a highly fit of the accumulating money to a linear trend. In specific, we have fitting equations and R-square values for each taste group below, in the order of how quick the money accumulated over trials. This finding is in line with the adjusted number of pump earlier.

Sour: $y = 3468.9x + 3170.6 \quad R^2 = 0.9989$

Neutral: $y = 3237.3x + 2666.4 \quad R^2 = 0.9998$

Demographic of participants

In total, we had 27 participants in this study (5 males, 22 females, mean age 22.22 SD 3.96). Among them 21 participants recognized the taste.

Table S 15. Average ratings (with standard error) of participants' preferences of each taste.

	Sweet Rating	Sour Rating	Salty Rating	Bitter Rating	Umami Rating
M	4.44	3.07	4.00	2.41	4.22
SE	0.16	0.22	0.16	0.23	0.24

Participants rated their preferences for each taste at the end of the experiment, after completing the BART task. Participants answered the questions "How much do you like the different tastes below?" on a scale from 1 to 6: "I don't like it at all", "I don't like it much", "Somewhat okay", "I mostly like it", "I like it very much", "I don't know". Table S15 below summarizes the mean ratings of all participants, and group by the stimulus groups (one of the five basic tastes that participants were assigned to). There was no bias of the participants' preference and the given taste.

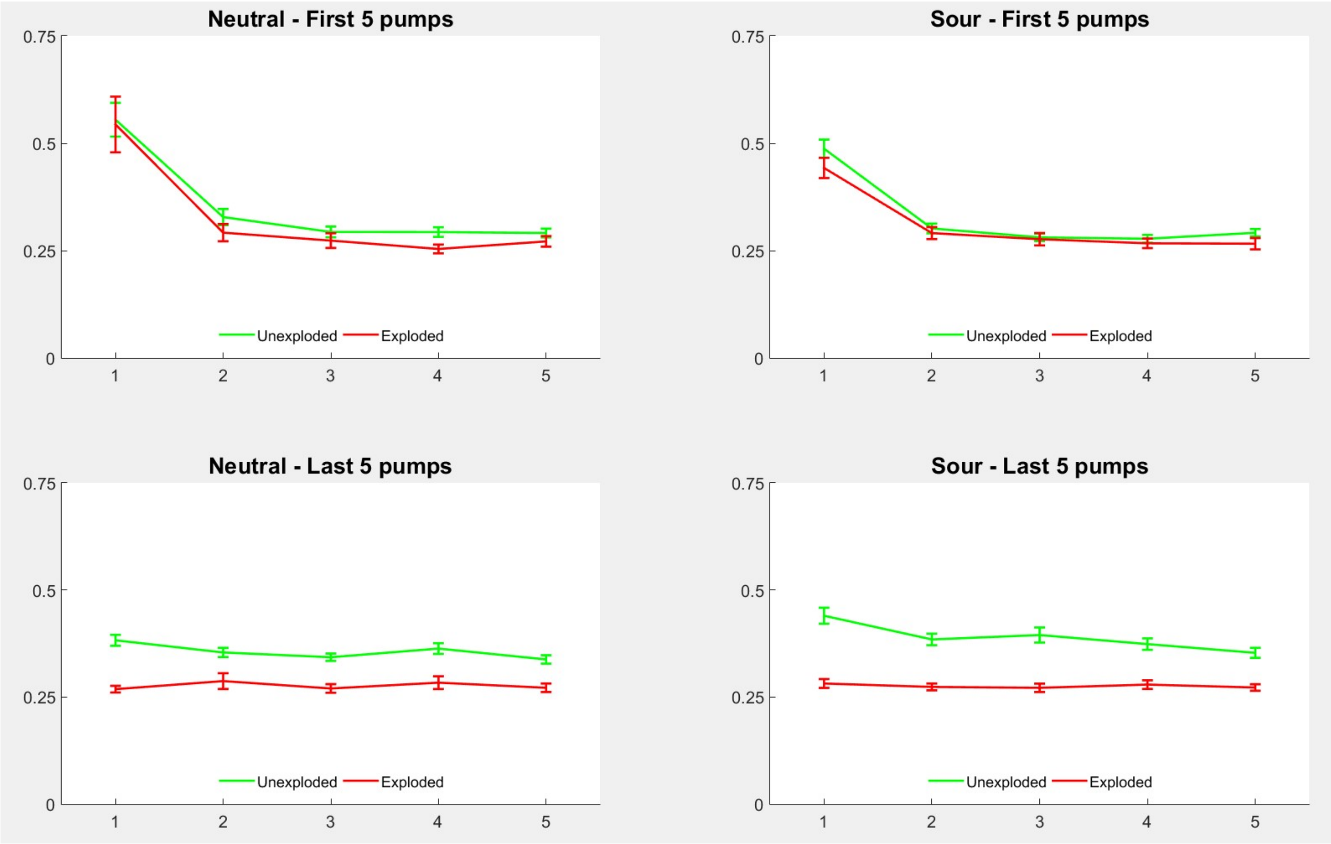

Figure S 27. Inter-click time of the first and last five pumps for each taste, between exploded and unexploded balloons (with standard errors). Bars represent standard error of the mean (SE).

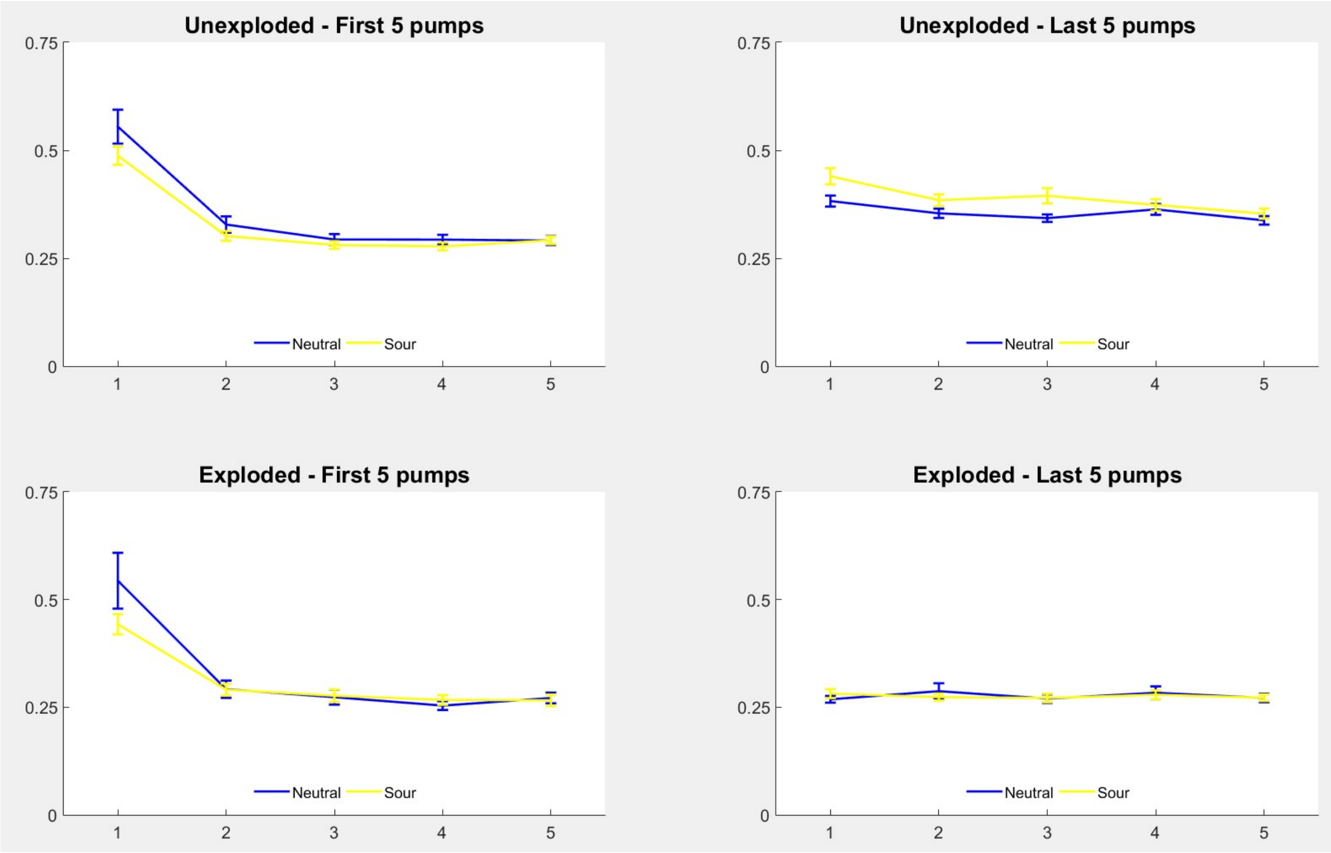

Figure S 28. Inter-click time of the first and last five pumps (with standard errors) for each stimulus, grouped by Unexploded balloons (top) and Exploded balloons (bottom). Bars represent standard error of the mean (SE).

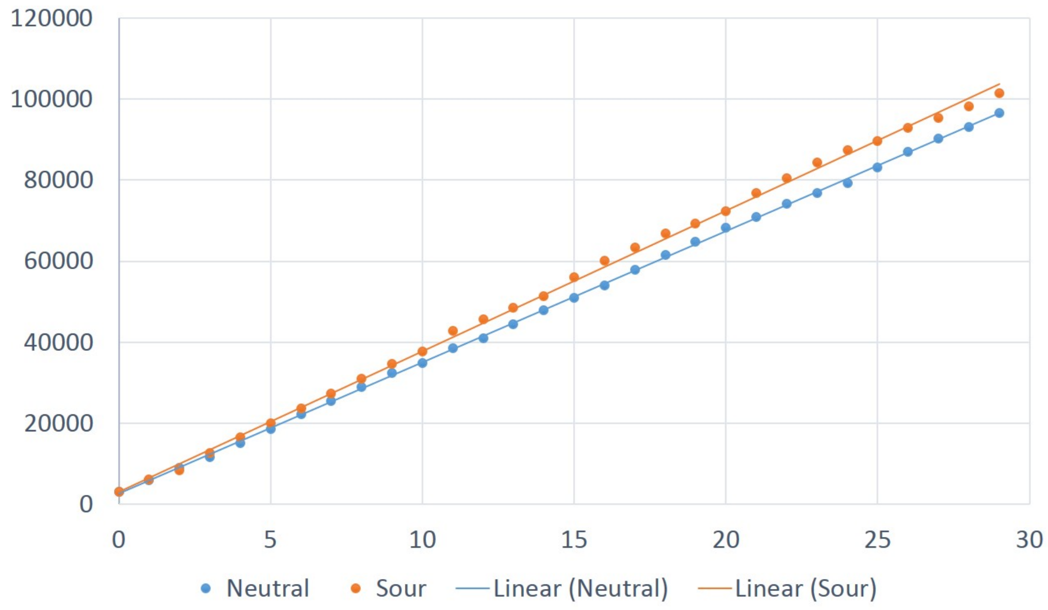

Figure S 29. Scatter plot of the average accumulate money after each trial/ balloon for sour and neutral groups, with the linear least-square fitting (vertical unit: points, horizontal unit: trial number).
